# Organic Waste-Based Fertilizer in Hydroponics Increases Tomato Fruit Size but Reduces Fruit Quality

**DOI:** 10.3389/fpls.2021.680030

**Published:** 2021-06-23

**Authors:** Dmitry Kechasov, Michel J. Verheul, Martina Paponov, Anush Panosyan, Ivan A. Paponov

**Affiliations:** ^1^Division of Food Production and Society, Department of Horticulture, Norwegian Institute of Bioeconomy Research (NIBIO), Ås Municipality, Norway; ^2^Department of Food Science, Aarhus University, Aarhus, Denmark

**Keywords:** *Solanum lycopersicum*, closed soilless cultivation, sustainable production, biogas digestate, nutrient recycling, xylem sap, metabolomics, moving bed bioreactor

## Abstract

In regions with intensive agricultural production, large amounts of organic waste are produced by livestock animals. Liquid digestate from manure-based biogas production could potentially serve as fertilizer if integrated with closed horticultural irrigation systems. The aim of this experiment was to investigate how fertilizer based on liquid biogas by-products of pig manure digestion can affect the growth and production of tomato plants. Integration of a nitrification bioreactor presumes a significantly lower concentration of nutrient solutions and a higher level of oxygenation than classical mineral cultivation. Therefore, additional controls were included. We compared plant growth and fruit quality traits of tomato plants grown in a hydroponic solution with organic fertilizer with two levels of mineral fertilizer. The tomatoes grown with organic waste-based liquid fertilizer showed reduced growth rates but increased mean fruit size, resulting in no significant change in total yield compared with high-mineral cultivation. The growth rate was similarly reduced in plants cultivated with low-mineral fertilizer. Plants cultivated with organic waste-based fertilizer had high Cl^−^ concentration in xylem sap, leaves, and, ultimately, fruits. The leaves of plants cultivated with organic waste-based fertilizer contained higher concentrations of starch and soluble carbohydrate and low concentrations of phosphorous (P) and sulfur (S). The plants grown with organic waste-based or low-mineral medium showed significantly poorer fruit quality than the plants cultivated with the high-mineral solution. The low-mineral treatment increased xylem sap contribution to fruit weight because of higher root power. The organic waste-based fertilization did not change the root power but increased fruit size. In conclusion, organic waste-based cultivation is a possible solution for sustainable plant production in greenhouses. However, additional adjustment of nutrient supply is required to improve fruit quality.

## Introduction

In northern latitudes, year-round cultivation of high-value vegetables, such as tomatoes, can only be conducted in greenhouses. In modern greenhouses, tomatoes are grown in soilless cultures using nutrient film techniques (NFT) or rockwool slabs with drip irrigation. Nutrient composition and supply are highly controlled to reduce fertilizer usage and water consumption. However, the average leaching rate from open drip irrigation systems is between 20 and 40% (Van Os, 1999). Closed-cycle soilless systems, where all leachate is recycled, can further reduce fertilizer run-off by 30–40% compared with open-cycle systems (Montesano et al., 2010).

Recycling soilless systems are used in many countries. However, these systems increase the risks of disease spread and investment costs for the construction of recycling facilities and leachate disinfection. In addition, current disinfection procedures (e.g., heat treatment, ozone, hydrogen peroxide, and UV radiation) are not sustainable (Maessen and Verheul, 2016). These treatments also create a sterile root environment, making roots prone to invasive diseases and increasing potentially explosive disease outbreaks. In contrast, experiences with organic growth systems have shown that the development of diseases is seldom explosive in these systems. This might reflect the natural non-sterile biological environment that promotes a root zone that is more resistant to invasive diseases.

One potential source of organic nutrients for soilless cultivation is organic wastes produced by livestock animals. The annual world production of nitrogen (N) fertilizer as livestock animal manure is 125 billion tons, but 70% of it is left on pastures and cannot be collected. The remaining amount of this potential N-fertilizer could theoretically supplement the yearly consumption of mineral N-fertilizers in the world (11 million tons) (FAOSTAT, 2019, http://www.fao.org/faostat/). Unused or misused fertilizers can also contribute to air pollution, acidification of soil, and eutrophication of nearby aquatic ecosystems (Carpenter et al., 1998; Mallin and Cahoon, 2003; Woodward et al., 2012; Mateo-Sagasta et al., 2017). The use of organic waste-based fertilizer in a closed recirculation system in a greenhouse would, therefore, be beneficial for the reduction of N and P pollution (Martinez-Alcantara et al., 2016) and carbon emission (Favoino and Hogg, 2008).

The main challenges that currently hinder the application of organic waste-based fertilizer in tomato greenhouse systems are difficulties that arise in providing the proper level and balance of nutrients, and dealing with the presence of phytotoxic organic compounds, heavy metals, and salts (Ehret et al., 2005; Jones, 2007). The effects of phytotoxic stress factors can be alleviated in part by oxygenation (van Os et al., 2012). However, little is known regarding the effects of organic waste-based fertilizer on the development of tomato plants and quality of tomato fruits. In other crops, organic fertilization may increase root development, for example, citrus trees grown in soil (Martinez-Alcantara et al., 2016), maize seedlings (Canellas et al., 2002; Jindo et al., 2012), and lettuce in hydroponic culture (Shinohara et al., 2011). Generally, tomato plants cultivated organically (e.g., in organic waste or manure) have comparable (Verheul, 2005; Mitchell et al., 2007; Shinohara et al., 2011; Antonious et al., 2019) or slightly lower (Zhai et al., 2009) yields than plants cultivated with conventional fertilizer. However, reductions in fruit size have often been reported (Oliveira et al., 2013; Zhang et al., 2016). This suggests that the choice of organic material may be important to ensure efficient tomato production.

Hydroponic cultivation of tomatoes in greenhouses could utilize many sources of liquid organic waste by-products, such as compost, biogas effluent, soluble fish waste, or corn steep liquor (Liedl et al., 2006; Zhai et al., 2009; Shinohara et al., 2011). The liquid effluent from anaerobic digestion (digestate) also has the potential for use as a fertilizer in hydroponic cultures (Cheng et al., 2004). During anaerobic digestion, nutritionally interdependent communities of bacteria and Archaea convert biomass into energy-rich biogas and nutrient-rich liquid digestates in the absence of oxygen (Sarker et al., 2019). The resulting digestate has a high concentration of ammonium (${\text{NH}}_{4}^{+}$) and potassium (K^+^) ions and can be readily generated from locally collected household wastes or livestock manure. Since digestate is a by-product of biogas production, it can be integrated into a sustainable energy production chain to minimize waste fraction.

In areas with both greenhouse and livestock productions, liquid organic waste from livestock can be used as a fertilizer for closed soilless cultivation of greenhouse crops. Soilless cultivation requires significantly less disinfection and does not destroy the natural microbial biodiversity in the rhizosphere (Sonneveld and Voogt, 2009).

The main challenge that limits the application of these fertilizer sources is N availability (Möller and Müller, 2012), as the bulk of N in organic fertilizers occurs in organic (org-N) or reduced (${\text{NH}}_{4}^{+}$-N) forms. Organic N occurs mostly in the form of urea and peptides. Peptides are largely unavailable for plants, while urea can be hydrolyzed to ammonium in digesters and in soils. Ammonium ions are soluble and easily taken up and utilized by plants, but they are harmful to plants in large concentrations (Magalhaes and Wilcox, 1984). Moreover, high levels of ammonium may reduce calcium uptake, thereby inducing disorders like blossom-end rot and subsequent losses in production (Hagassou et al., 2019). Deficiencies in nutrients like N in organic waste-based fertilizers can be overcome by the addition of mineral fertilizer, as has been shown for tomatoes (Poustkova et al., 2009).

The bioavailability of N for plants can also be improved by oxidation of reduced forms of N to nitrate *via* nitrification. This process naturally occurs in soil, mainly because of the activity of aerobic bacteria. In soilless cultures, bioreactors hosting nitrifying bacteria can be integrated into recirculation systems to provide plants with N (Shinohara et al., 2011; Saijai et al., 2016) with added benefit of reducing risk of pathogen infection. Moving bed biofilm reactors (MBBRs) use a large surface area in combination with aeration to produce bacterial sludge that can convert ammonia to nitrate (Rusten et al., 2006). Aerobic conditions may also help to mineralize org-N from organic waste-based fertilizers (Möller and Müller, 2012). Thus, a closed recirculation system with built-in nitrification bioreactor can be viewed as a reasonable setup for the cultivation of vegetables in a greenhouse.

The use of organic waste as a fertilizer can also create salinity problems, especially in closed irrigation systems. Salt toxicity may influence the uptake of nutrients, especially Ca^2+^ and K^+^ ions, thereby reducing the commercial quality and yield of tomatoes and negatively affecting the growth and photosynthetic efficiency of tomato plants (Sonneveld and Voogt, 2009). Thus, a closed recirculation system with built-in nitrification bioreactor can be viewed as a reasonable set up for the cultivation of vegetables in a greenhouse.

Although many different types of organic waste can potentially be used to grow tomatoes in soilless culture, the long-term effects of organic waste-based fertilizers on plant growth, development, and fruit quality have not been sufficiently investigated. Moreover, no studies have examined how the cultivation of tomatoes in soilless cultures using fertilizers based on organic wastes might affect the nutritional quality of their fruits.

We have analyzed nutrient content, quality, and growth of tomatoes cultivated in an industrial greenhouse using a closed irrigation system with organic waste-based fertilizer. We have estimated the effect of organic waste-based fertilizer on root activity, evaluated root pressure by measuring xylem sap exudation, and analyzed the xylem sap composition of ions and metabolites.

The objective of this study was to investigate the effect of a fertilizer derived from the liquid effluent of a pig manure-based biogas production system on the physiology of greenhouse tomato plants and the quality of their fruits.

- The taste quality and yield of tomatoes cultivated with organic waste-based fertilizer (EC 0.5 ± 0.2 mS·cm^−1^) were compared with tomatoes grown with mineral fertilizer in low (EC 0.4 ± 0.2 mS·cm^−1^) and high concentrations (EC 3.8 ± 0.6 mS·cm^−1^).
- The effect of different types of fertilizer on the size of tomato fruits was investigated.
- The differences in plant development, biomass distribution, and photosynthetic ability between plants grown with different fertilizers were investigated.
- The effect of different fertilizers on root metabolism was investigated by the analysis of xylem sap composition.

## Materials and Methods

### Plant Materials and Growth Conditions

**Figure 1:**
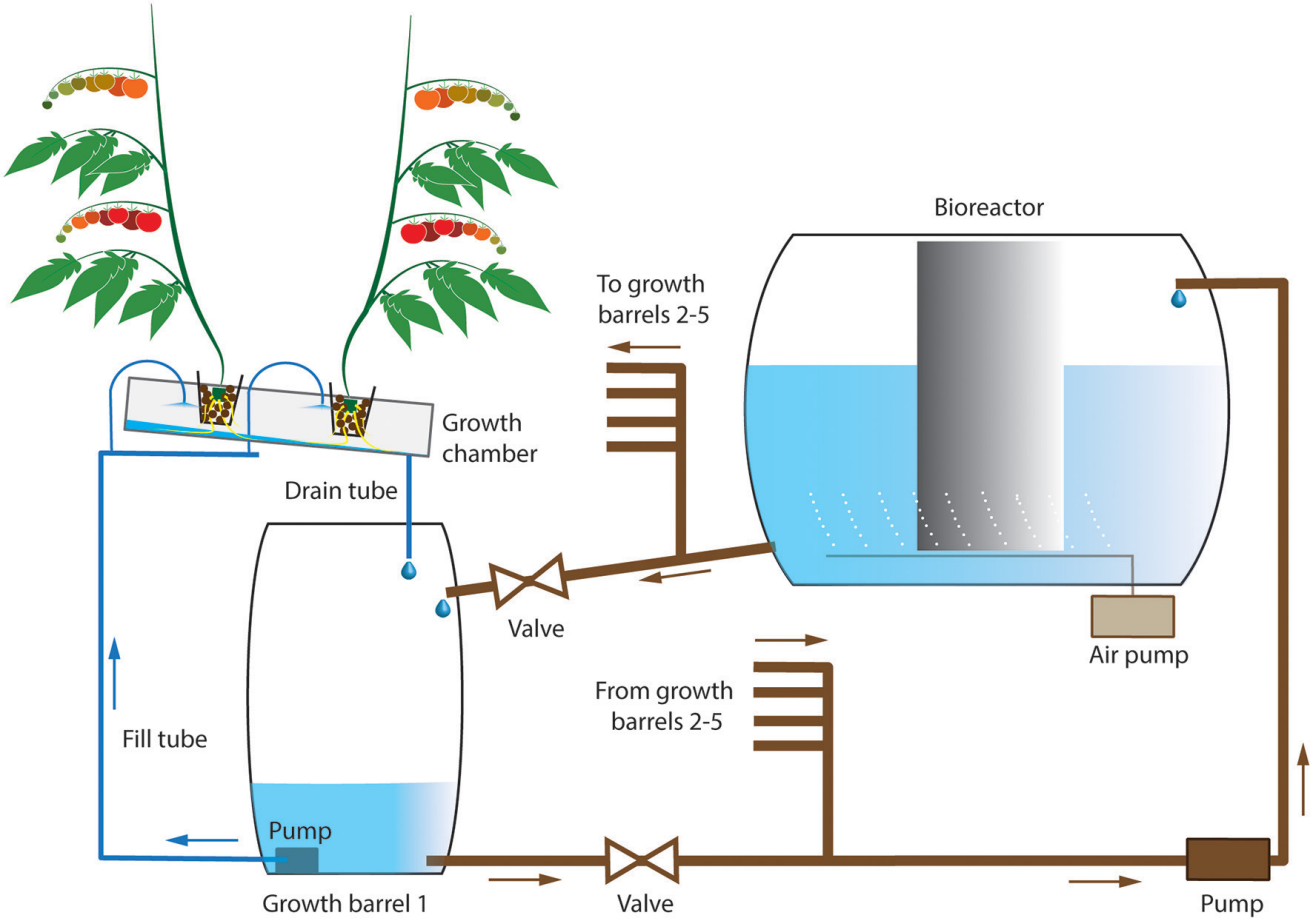
Experimental setup for the organic waste-based and low-mineral treatments. The nutrient solution was mainly contained in a large reservoir (e.g., bioreactor) and gravimetrically transported to each of five growth barrels. The nutrient solution from each growth barrel was pumped into the growth chamber and sprayed on roots. The nutrient solution returned to the barrel through the drain pump, and was collected from all five pumps and returned to the large reservoir. For the high-mineral treatment, the large barrel was used instead of the bioreactor. The plants in rockwool cube were placed in expanded clay pebbles.

Tomato plants (*Solanum lycopersicum cv*. Dometica) were cultivated in a Venlo-type greenhouse in southwestern Norway (58°42'49.2“N 5°31'51.0”E) from September 21, 2018 to January 22, 2019. Seeds were sown on August 13, 2018, initially grown in a growth chamber in rockwool cubes (Grodan, Roermond, the Netherlands) and watered daily with high-mineral fertilizer with electrical conductivity of 3.2 mS cm^−1^. For climate adaption, plants were transported into the greenhouse and grown for three weeks prior to transplanting them into AeroFlo growing chambers (General Hydroponics, Santa Rosa, CA, United States). In the growing chambers, the plants in rockwool cubes were fixed with expanded clay pebbles inside a plastic mesh pot. A nutrient solution was sprayed on the mesh pots, and the plant roots were not submerged in the nutrient solution until they reached the bottom of the growth chamber (Figure 1). The tomatoes were cultivated as one-shoot plants in a high-wire system.

High-pressure sodium (HPS) lamps (Philips GP Plus 750 W, Gavita Nordic AS, Andebu, Norway) with the intensity of 161·W m^−2^ were installed about 1.5 m above the top of the canopy. The threshold for turning on HPS lights was set to 250 W·m^−2^ of incoming solar radiation, which means that the HPS lamps were switched on continuously for 12 h per day for weeks 38–41, then for 15 h for week 42, and 18 h from week 43 to the end of the experiment. During the experiment, the following environmental conditions were measured in the greenhouse: average daily temperature of 21.3 ± 2.4°C, relative humidity of 68 ± 13%, the CO_2_ concentration of 682 ± 209 ppm, and solar radiation of 30 ± 72 W·m^−2^. Tomato flowers were pollinated by bumblebees. The plants were lowered weekly by about 30 cm. Side shoots were removed, and three or more leaves were removed below the truss that first showed orange tomatoes. The trusses were pruned to seven fruits per truss.

### Experimental Setup

The set up consisted of three closed irrigation systems, organic waste-based, low mineral, and high mineral. The first two systems included an MBBR. Each treatment was represented by one main reservoir with nutrient solution (550 L biofilter or 220- L barrel), five small reservoirs (60 L barrels), each barrel was connected to an AeroFlo growth chamber with two tomato plants (Figure 1). The total number of plants per treatment was 10 (*n* = 10). We selected low-pressure hydroponics over drip irrigation because of (1) elimination of clogging due to sludge/particle buildup in hoses and rockwool slabs and (2) the possibility to investigate intact root architecture and take samples.

The main reservoirs were placed outside of the greenhouse compartment and were connected to the small reservoirs through a system of hoses and pipe manifolds (Figure 1). The nutrient solution flowed gravimetrically from the main reservoir to the small reservoirs and was pumped back by a water pump. A small pump was used to transport the nutrient solution to the AeroFlo growing chambers located above the small reservoirs. The nutrient solution was sprayed continuously onto the roots. The growing chambers were placed at an angle to allow the nutrient solution to return to the small reservoirs through a drain tube located at the lowest point of the growth basket (Figure 1). In other words, the nutrient solution circulated simultaneously between the main and small reservoirs, and between the small reservoirs and the greenhouse.

Fertilizer was added after regular monitoring of nitrate level two to three times per week with the help of semi-qualitative test strips (Quantofix, 91313, Sigma-Aldrich, Darmstadt, Germany). The minimum nitrate level in the nutrient solution was set to 1 mmol·L^−1^. The plants in the control treatment (i.e., high-mineral) received a complete nutrient solution based on standardized recommendations containing the following: 17.81 mM NO${}_{3^{-}}$, 0.71 mM ${\text{NH}}_{4^{+}}$, 1.74 mM P, 9.2 mM K, 4.73 mM Ca, 2.72 mM Mg, 2.74 mM S; and micronutrients: 15 μmol Fe, 10 μmol Mn, 5 μmol Zn, 30 μmol B, 0.75 μmol Cu, and 0.5 μmol Mo. The electrical conductivity of the nutrient solution was about 3.5 mS·cm^−1^, and the pH was 5.9. The same stock nutrient solution was added to the low-mineral treatment as fertilizer. Its initial concentration was ~20-fold lower than that of the high-mineral treatment. Tap water was used to compensate for plant water uptake. In the organic waste-based treatment, the liquid digestate was used as the main fertilizer. However, some mineral salts (Ca, Mg, P, and S) were added to correct for nutrient unavailability. The initial physiochemical parameters and ionic composition of all tested treatments can be found in Supplementary Table 1. Nitric acid (HNO_3_) was used to adjust the pH to about 7 to ensure bacterial growth. A mixture of essential microelements Pioneer red (Yara, Oslo, Norway) was also added to the organic waste-based treatment before transferring the plants (4.5 ml) and 56 days after transferring (6 ml).

Liquid digestate was produced from pig manure by anaerobic mesophilic process (average reactor temperature 37°C, filtration with 200-micron filter, 10 days hold time). The digestate was autoclaved at 120°C for 15 min, and its pH was 7.5 before autoclaving and 9.7 after autoclaving, EC 2.7 mS·cm^−1^. The average chemical oxygen demand (COD) of the digestate was 5.4 ± 0.8 g·L^−1^, total solids (TS) 8 ± 0.7 g·L^−1^, and volatile solids (VS) 52 ± 4%. The digestate was diluted ~20 times and was stored indoors at 7–15°C in closed plastic barrels.

An MBBR (Nexus220, Evolution Aqua, Wigan, United Kingdom) was used for biological nitrification of biogas effluent. The biofilter was 1 m in diameter and had a water depth of 1 m, and it contained 150 L of K1 micro Kaldnes media (Evolution Aqua, Wigan UK) with a specific surface of 950 m^2^·m^−3^. The digestate was manually added into the inner chamber, where it was mixed with recycled nutrient solution collected from the small barrels and mechanically filtered. A well-mixed solution was passed to the outer chamber containing Kaldnes media with resident nitrifying bacteria. The nitrified solution was then gravimetrically transported into the greenhouse for fertigation. Organic waste-based treatment had the following characteristics (*n* = 4): soluble solids (SS) 86 ± 28 mg·L^−1^, TS 605 ± 127 mg·L^−1^, and VS 79 ± 10%.

Physiochemical parameters of the nutrient solution (temperature, pH, EC, etc.) were monitored two times each week with a multimeter (HI 98194, Hanna Instruments, Woonsocket, RI, United States). Samples of the nutrient solution were taken every week from the main reservoir before adding fertilizer and frozen at −20°C until analysis.

### Harvest

Fruits were harvested two times per week. The harvested fruits were weighed individually, and their position in the truss and the number of trusses were recorded. Each position for every treatment was represented by 47–70 fruits. Final harvests were performed on February 22 and 24, 2019. To estimate dry matter content, leaves, stems, and fruits were completely dried at 70°C (~2 weeks for fruits, otherwise 96 h).

### Xylem Sap Collection

Xylem sap was collected on February 22 and 24, 2019 from four randomly selected shoot plants using a root pressure method adapted from Alexou and Peuke (2013). Stems of selected plants were cut with garden scissors at a position about 10 cm above the root-shoot interface. The cut surface was cleaned with deionized water, and a silicon tube was fixed over the stump and sealed with silicone grease. The exudate collected from the silicone tubes during the first 60 min period was discarded with a pipette. The xylem exudate collected during the next 60 min period was stored in plastic vials on ice and subsequently frozen in liquid N, and stored at −80°C.

### Quality Analysis of Fruits

Samples for fruit quality assessment were collected on January 1, 2019 (harvest 1), January 9, 2019 (harvest 2), and January 21, 2019 (harvest 3). Each replicate consisted of six tomato fruits selected from a pool of fruits collected from 10 plants per treatment. To ensure that the tomatoes had equal ripeness, tomato fruits were sampled at grade 8, determined visually based on a scale provided by Bama AS (Oslo, Norway) (from 1, green to 12, deep red).

For each harvest, three replicates (*n* = 3) per treatment were investigated for firmness, SS content (SSC), and total titratable acidity (TTA). For one tomato, firmness was measured three times using a Durofel firmness tester (Agro Technologies, Forges-les-Eaux, France) and averaged on a scale from 1 to 100, where 100 meant full firmness and 1 meant complete lack of firmness (i.e., Durofel units). The tomatoes were then cut into four parts. One quarter from each of six fruits was combined to make a replicate (a total of six quarters) and homogenized with a handheld blender. The resulting homogenate was used for determination of SSC and TTA on the same day as harvesting, following the procedures published by Mitcham et al. (1996) and Verheul et al. (2015). The SSC (expressed as Brix) was measured with a digital PR-101α refractometer (ATAGO, Tokyo, Japan) set to zero with deionized water. The TTA was determined using 794 Basic Titrino (Metrohm, Herisau, Switzerland), an automatic titrator, with potentiometric detection (pH_final_ 8.2) and expressed percent of citric acid equivalents (CAE) per 100 g FW (Verheul et al., 2015). An aliquot of tomato fruit homogenate was transferred into a 1.5-ml centrifuge tube, weighed, immediately frozen in liquid N, and stored at −80°C until further.

### Analysis of Soluble Ions

The ion composition analysis of the nutrient solution, fruits, and xylem sap was performed by using ion chromatography with conductive detection, as described in Paponov et al. (2020).

### Leaf Area Measurements

The fresh weights of proximal leaflets of leaves at the bottom of the canopy were measured, and the area was determined using LI-3100 Area Meter (LI-COR, Inc., Lincoln, NE, United States). The leaves were dried at 65°C for 48 h.

### Extraction and Separation of Polar and Non-polar Metabolites From Leaves

The extraction of metabolites from leaves and their separation were performed using a two-phase separation method described by Salem et al. (2016). In short, distal leaflets of mature undamaged leaves at the bottom of the canopy were collected at the end of the day (ED, i.e., 12 midnight) and the end of the night (EN, i.e., 6 a.m.). The leaves were immediately frozen in liquid N and stored at −80°C. Prior to the analysis, leaf samples were lyophilized for ~48 h without heating in freeze dryer BK-FD10S (BIOBASE, Jinan, China). Thereafter, the leaves were ground in a grinding mill (Star-Beater, VWR, Radnor, USA) in a pre-cooled holder using pre-cooled stainless-steel balls (0.5 g) for 2 min at max frequency (30 Hz). The ground samples were pre-cooled with liquid N spatulas and transferred in new 2 ml centrifuge tubes. Approximately 20 ± 0.5 mg of the dried leaves were aliquoted in the new tubes and used for sample extraction. In each tube, 1 ml of a pre-cooled mixture of methyl *tert*-butyl ether (MTBE) and methanol (MeOH) in proportion 3:1 (v/v) was added. The tubes were incubated in an orbital shaker at 100 rpm for 45 min at 4°C. Thereafter, the tubes were sonicated for 15 min in an ice-cooled sonication bath (USC300TH, VWR, USA). To initiate phase separation, 650 μl of H2O:MeOH (3:1, vol/vol) was added to each sample tube. The mixture was vortexed for 1 min (Vortex Genie 2, Scientific Industries, Inc., Bohemia, USA) and spinned in a centrifuge (MicroStar 17R, VWR, USA) at 17,000 × g for 15 min at 4°C, and 450 μl of the solvent from the upper, non-polar, phase was transferred into a 1.5-ml microcentrifuge tube for pigment analysis. The remaining upper phase and interphase were discarded, and 400 μl of the solvent from the lower phase containing polar and semi-polar metabolites was transferred into a new 1.5-ml microcentrifuge tube. The extracts were used directly or stored at −80°C. For further starch analysis, the remaining liquid was carefully removed by pipetting. The obtained pellet was washed with 500 μl MeOH by vortexing for 1 min. The samples were centrifuged at 10,000 × g for 5 min at 4°C.

### Quantification of Glucose, Fructose, and Sucrose

The quantification of glucose, fructose, and sucrose was performed by sequential enzymatic assays with photometric detection in a spectrophotometric plate reader (Multiscan GO, Thermo Fisher Scientific, Waltham, USA) according to Zhao et al. (2010).

To estimate glucose concentration, 10 μl of the polar solvent phase was diluted eight times with 80% ethanol in a 1.5 centrifuge tube, and 20 μl of the resulting mixture was transferred into a 96-well-plate in triplicate. The ethanol was evaporated from the wells in an oven at 60°C for ~40–50 min. The dried material was resuspended by the addition of 20 μl of deionized water, then 100 μl of the glucose hexokinase (HK) assay reagent (G3293, Sigma) was added into each well. The 96-well-plate (UV-STAR, Greiner Bio-One, Kremsmünster, Austria) was covered with a lid and incubated inside the plate reader for 15 min at 30°C. The absorbance of samples, blanks, and standards was measured at 340 nm at 30°C and in precision mode.

The amount of fructose was determined by phosphoglucose isomerase (PGI) assay, and 10 μl of the PGI assay reagent (0.2 M HEPES with pH 7.8) was added into each well previously used for glucose quantification. The absorption was measured at 340 nm after incubation inside the spectrophotometer for 15 min at 30°C.

The amount of sucrose was determined by adding 10 μl of the invertase assay reagent (10 mg ml^−1^, I4504, 300 units·mg^−1^, Sigma) in 1 M Na-citrate buffer with pH 6) into each well. Absorption was measured at 340 nm after incubation inside of the spectrophotometer for 60 min at 30°C.

### Extraction of Starch

The analysis of starch content was performed using the remaining pellet after liquid extraction according to Salem et al. (2016) with modifications, and 1 ml of 80% ethanol was added to each tube. The tubes were incubated for 3 min at 80°C in a water bath and then cooled at RT. The insoluble material was spinned down (5 min, 10,000 × g, RT). By the end of the third washing step, the pellet had a light brown color. The samples were placed in a chemical hood to allow ethanol to evaporate. The dried pellet was resuspended in 0.5 ml of deionized water and vortexed for 1 min for homogenization. The starch was gelatinized at 100°C for 15 min in the water bath and vortexed for 1 min, and 200 μl of homogenate pellet was transferred to each of two 1.5-ml microcentrifuge tubes, containing: (a) 200 μl of digestion mix and 200 μl of buffer solution (200 mM sodium acetate–acetic acid, pH 4.8). The digestion mix contained 6 U·ml^−1^ of α-amyloglucosidase from *Aspergillus niger* (6 U·mg^−1^11202367001, Roche, Basel, Switzerland) and 10 μl per 20 mg sample DW of α-amylase (A4582, Sigma). The tubes were incubated at 60°C for 30 min in the water bath, and then centrifuged at 14,000 × g for 10 min at RT to remove undigested material. The glucose content from starch was quantified using the previously described HK enzymatic assay. Starch concentration was determined by multiplying glucose concentration by dilution factor and by the glucose equivalent of 0.9 (Chow and Landhausser, 2004; Hostettler et al., 2011).

### Analysis of Pigments

Chlorophyll (a and b) and carotenoid content in the tomato leaves was determined spectrophotometrically. A 35- μl volume of the upper polar phase was diluted 20 times with MeOH, and 200 μl of the diluted mixture was pipetted in triplicate into a 96-well-plate. Absorbance was measured at 470, 652, and 665 nm. The concentration of carotenoids was measured using the formula for pure MeOH from Lichtenthaler and Buschmann (2001). Path length correction factors for the 96-well-plate were measured and applied as specified by Warren (2008). The concentration of chlorophylls a and b, and total carotenoids was calculated as mg·g^−1^ DW.

### Total N Determination

The total N content of the tomato leaves was determined by using persulfate digestion (Purcell and King, 1996; De Borba et al., 2014) coupled with ion chromatography. Approximately 20 mg of lyophilized leaves were mixed with 4 ml of a digestion mixture (0.475 M NaOH and 0.167 M K_2_S_2_O_8_) in glass centrifuge tubes and closed with Teflon-lined caps. The tubes were incubated in a heating block at 120°C for 60 min. The N content was determined by suppressed ion chromatography. Elution parameters were similar to those previously described (Paponov et al., 2020), except that acetone was not used for eluent preparation to avoid peak coelution. Recovery of N extraction was 78% for inorganic ammonium salt and glycine standards. Organic N was calculated by subtraction of inorganic N (NH_4_-N, NO_2_-N, and NO_3_-N) from the total N concentration. Values of N materials were presented as g·100 g^−1^ leaf DW.

### Metabolic Analysis of Xylem Sap

The extraction of polar metabolites was performed according to Fiehn (2003). In short, frozen xylem sap was thawed, and 100 μl of it was added to water:chloroform mixture (500 μl water and 300 μl chloroform with 10 μl ribitol standard with concentration 0.1 mg·ml^−1^). The mixture was vortexed for 30 s and centrifuged at 17,000 × g for 2 min at 4°C, and 450 μl of the upper phase was transferred into a new 1.5-ml tube and lyophilized overnight.

Metabolite derivatization was performed according to Hill and Roessner (2013) with minor modifications. After lyophilization, the samples were quickly spinned, and 40 μl of methoxamine in pyridine (20 mg·ml^−1^) was added. The tubes were incubated for 90 min at 37°C with a shaking speed of 750 rpm. Thereafter, 80 μl of N-Trimethylsilyl-N-methyl trifluoroacetamide (MSTFA, 394866, Sigma-Aldrich) containing a mixture of alkanes (C8-C40, 40147-U, SUPELCO, Sigma-Aldrich) for retention index calculation was added, and the tubes were incubated for 30 min at 37°C with the same shaking speed. After incubation, the tubes were centrifuged for 1 min at 17,000 × g at RT, and 50 μl of the sample was transferred into a GC vial with a micro insert and closed with a screw cap containing Teflon septa.

Derivatized samples were immediately transferred to a GC instrument. The samples were analyzed with Agilent 6890 Gas Chromatograph coupled with Agilent 5975 Mass Selective Detector (MSD, Agilent Technologies, Santa Clara, CA, United States). Chromatographic separation was performed on Rxi-5Sil MS with an Integra-Guard column (30 m long, 0.25 mm inner diameter, 0.25 μm film thickness, Restek, Bellefonte, PA, United States), and 1 μl of the sample was automatically injected by the MPS autosampler (Gerstel, Mülheim, Germany) equipped with a 10-μl syringe (Gerstel, Germany) in a pulsed splitless injection mode at 230°C. Pulse was held at 200 kPa for 2 min. Helium was used as a carrier gas at a constant flow rate of 1 ml·min^−1^. The temperature of the GC oven was ramped from initial 70°C to final 325°C at rate 5°C·min^−1^ and held at final temperature for 3 min (54 -min run in total). MSD was tuned with perfluorotributylamine before the analysis. The MSD was operated in electron ionization mode at 70 -eV electron ionization energy. The temperatures of the transfer line, the ion source, and the quadrupole were set to 280, 230, and 150°C. Mass spectra were recorded at frequency 5.5 scans/s with a scanning range from 50 to 550 m/z.

Data acquisition was carried out by MassHunter GC-MS software (version B.07.00, Agilent Technologies, Santa Clara, CA, United States). Data analysis was performed with the MS-DIAL software version 4.38, compound identification was performed using an integrated compound library (GCMS DB_AllPublic-KovatsRI-VS2) using both spectrum and retention index similarity (Tsugawa et al., 2015: Lai et al., 2018). Peak heights present in blank samples were subtracted from corresponding peak heights in samples. Normalization (sum of peak heights of identified metabolites, i.e., mTIC), data filtering [default setting for interquartile range (IQR) filter] transformation (log and Pareto scaling), and statistical analysis [ANOVA, heatmap, principal component analysis (PCA), and sPLS-DA] were performed in MetaboAnalyst version 5.0 (Chong et al., 2019).

### Statistics

Each treatment consisted of 10 plants grown in pairs. Biomass accumulation traits of above-ground parts (plant length, number of trusses, leaves, etc.) were collected individually for each plant (*n* = 10). The roots of the plants grown in pairs were interconnected, and their weight was determined pairwise (*n* = 5). Total biomass accumulated during growth was calculated for each plant pair. Leaf samples for chemical analysis were collected from two plants grown in pairs (*n* = 5). Fruit numbers and their weight were recorded for each plant. The quality analysis of fruits was performed with fruits pooled from different plants within the same treatment. Statistical differences were evaluated using the general linear model (ANOVA) followed by Tukey's multiple comparisons test using the Minitab 19 software. Datasets were tested in the presence of normal distribution and identified outliers were removed. The level of significance was set as 0.05 (*p* = 0.05). Data were presented as mean values with SD.

## Results

### Physiochemical Parameters of the Nutrient Solution

The physiochemical parameters of the nutrient solution and its ionic composition were monitored weekly for the entire cultivation period. The average values obtained during cultivation are presented in Table 1, Supplementary Figure 1, Supplementary Table 1.

**Table 1:** Physiochemical parameters and ionic composition of the tested treatments.

**Fertilizer**	**Organic waste-based**	**Low-mineral**	**High-mineral**
**Physiochemical parameters** ***n=*** **28–31**			
Temperature (°C)	18.4 ± 2.3^a^	18.4 ± 2.3^a^	17.1 ± 2.3^a^
ORP (mV)	273 ± 57^a^	271 ± 40^a^	291 ± 44^a^
DO (% saturation)	83.4 ± 5.5^a^	83.2 ± 6.2^a^	63.5 ± 15.5^b^
pH	6.3 ± 1.0^a^	7.0 ± 0.5^b^	6.4 ± 0.3^a^
EC (mS·cm^−1^)	0.5 ± 0.2^a^	0.4 ± 0.2^a^	3.8 ± 0.6^b^
Water use (L·plant^−1^·day^−1^)	1.02	1.33	0.83
**Cations (mmol·L**^**−1**^**)** ***n*** **=** **17**			
Na^+^	1.5 ± 0.6^a^	0.2 ± 0.1^b^	0.4 ± 0.2^b^
${\text{NH}}_{4}^{+}$	0.181 ± 0.334^ab^	0.003 ± 0.007^b^	0.370 ± 0.479^a^
K^+^	0.79 ± 0.63^a^	0.28 ± 0.44^a^	10.36 ± 0.94^b^
Ca^2+^	0.42 ± 0.11^a^	0.91 ± 0.46^a^	7.74 ± 3.39^b^
Mg^2+^	0.2 ± 0.08^a^	0.34 ± 0.18^a^	3.95 ± 1.28^b^
**Anions (mmol·L**^**−1**^**)** ***n*** **=** **18–19**			
Cl^−^	1.05 ± 0.19^a^	0.16 ± 0.2^b^	0.19 ± 0.19^b^
${\text{NO}}_{2}^{-}$	0.03 ± 0.03^a^	0.02 ± 0.01^a^	0.17 ± 0.22^b^
${\text{NO}}_{3}^{-}$	1.5 ± 1.1^a^	1.5 ± 1.1^a^	23.5 ± 5.4^b^
${\text{PO}}_{4}^{3-}$	0.28 ± 0.18^a^	0.20 ± 0.16^a^	1.52 ± 0.53^b^
${\text{SO}}_{4}^{2-}$	0.12 ± 0.12^a^	0.26 ± 0.11^a^	2.63 ± 0.72^b^

*ORP, oxidation-reduction potential; DO, dissolved oxygen; EC, electrical conductivity. Mean values ± sd are shown. Different letters indicate statistically significant differences between treatments at p = 0.05*.

The high-mineral treatment was a solution with high nutrient concentration typically used for hydroponic tomato cultivation. The organic waste-based treatment was characterized by significantly lower concentrations of nutrients in the solution because nutrient influx in this system occurs continuously because of digestion of organic compounds and nitrification by bioreactor. These conditions prevent the accumulation of nutrients at the high levels found in the high-mineral nutrient solution. We addressed the effects related to differences in ion concentrations between the high-mineral and organic waste-based treatments by including an additional control, consisting of diluted mineral concentrations (the low-mineral treatment) that mimicked the nutrient concentrations found in the organic waste-based treatment. This experimental design meant that the high-mineral solution had the highest concentration of major nutrients (Table 1) and therefore the highest conductivity (3.8 ± 0.6 mS·cm^−1^), whereas the organic waste-based and low-mineral solutions had conductivity values of 13 and 11% of the high-mineral solution, respectively (Table 1). The high-mineral solution was not aerated; therefore, the dissolved oxygen (DO) was significantly lower in the high-mineral treatment (63.5 ± 15.5 %) than in the organic waste-based and low-mineral treatments (83.4 ± 5.5 and 83.2 ± 6.2, respectively). None of the other monitored parameters differed significantly, except for pH, which was 7 in the low-mineral, 6.4 in the high-mineral, and 6.3 in the organic waste-based treatments (Table 1).

The organic waste-based treatment had 3.8- and 7.5-fold higher concentrations of Na^+^ than the high- or low-mineral treatment, respectively, and the concentration of Cl^−^ in the organic waste-based treatment was 5.5- and 6.6-fold higher than in the low-mineral and the high-mineral treatments, respectively (Table 1). The average concentrations of other major soluble inorganic ions (${\text{NH}}_{4}^{+}$, K^+^, Ca^2+^, Mg^2+^, ${\text{NO}}_{2}^{-}$, ${\text{NO}}_{3}^{-}$, ${\text{PO}}_{4}^{3-}$, and ${\text{SO}}_{4}^{2-}$) were similar for the organic waste-based and low-mineral treatments throughout the cultivation.

The estimated average water use for the organic waste-based, low-mineral, and high-mineral treatments was 1.02, 1.33, and 0.83 L·plant^−1^·day^−1^, respectively. This was equivalent to 79, 103, and 64% of the water used when growing tomato plants in rockwool slabs with drip irrigation and without recirculation.

The estimation of relative N distribution showed that the low-mineral treatment had the highest percentage of used N from the nutrient solution (74%, compared with 60% for organic waste-based and 46% for high-mineral treatments, Supplementary Figure 3).

### Fruit Yield and Tomato Size

The differences in plant nutrition affected the total plant yield, total number of fruits and trusses, and individual tomato fruit size (Table 2). The plants cultivated with low-mineral solutions had significantly lower yields than the plants grown with the high-mineral treatment. The plants grown with the organic waste-based treatment also had lower yields than the plants grown with the high-mineral solution. However, this difference was not statistically significant. The plants grown with the organic waste-based and low-mineral treatments had lower numbers of fruits compared to those grown with the high-mineral treatment (38.7 ± 10.1, 39.6 ± 9.7, and 54.4 ± 10.6, respectively), whereas the total number of trusses and number of trusses with ripened tomatoes were significantly different only between the low-mineral and high-mineral treatments (Table 2).

**Table 2:** Commercial yield and yield components of tomato grown under different nutrient conditions.

**Fertilizer**	**Yield per plant (kg·plant^**−1**^) *n =* 10**	**Yield per area (kg·m^**−2**^) *n =* 10**	**Mean fruit weight (g) *n =* 384-537**	**Number of fruits (fruits·plant^**−1**^) *n =* 10**	**Total number of trusses (truss·plant^**−1**^) *n =* 10**	**Number of trusses with ripen tomato (truss·plant^**−1**^) *n =* 10**
Organic waste-based	3.7 ± 1.3^ab^	15.9 ± 5.5^ab^	94.2 ± 22.1^a^	38.7 ± 10.1^a^	14.5 ± 1.3^ab^	6.3 ± 1.3^ab^
Low-mineral	3.5 ± 1.0^b^	15.0 ± 4.5^b^	87.7 ± 23.2^b^	39.6 ± 9.7^a^	13.7 ± 1.7^a^	5.8 ± 1.0^a^
High-mineral	4.7 ± 0.9^a^	20.286 ± 4.0^a^	86.2 ± 18.6^b^	54.4 ± 10.6^b^	15.8 ± 1.0^b^	7.5 ± 1.4^b^

*Mean values ± sd are shown. Different letters indicate statistically significant differences between treatments at p = 0.05*.

Plants that received the organic waste-based fertilizer had the highest fruit fresh weight compared with the high-mineral (+10%) or low-mineral (+8%) treatment. Significantly fewer fruits werev produced with both the organic waste-based and low-mineral treatments. The number of fruits in a plant is dependent on the number of developed trusses, indicating that low nutrient supply might be the main factor delaying plant development. However, the organic waste-based treatment showed a weak tend toward a higher number of trusses than the low-mineral treatment, indicating that some other factor common only to the organic waste-based treatment might partly restore plant development under low nutrient availability.

**Figure 2:**
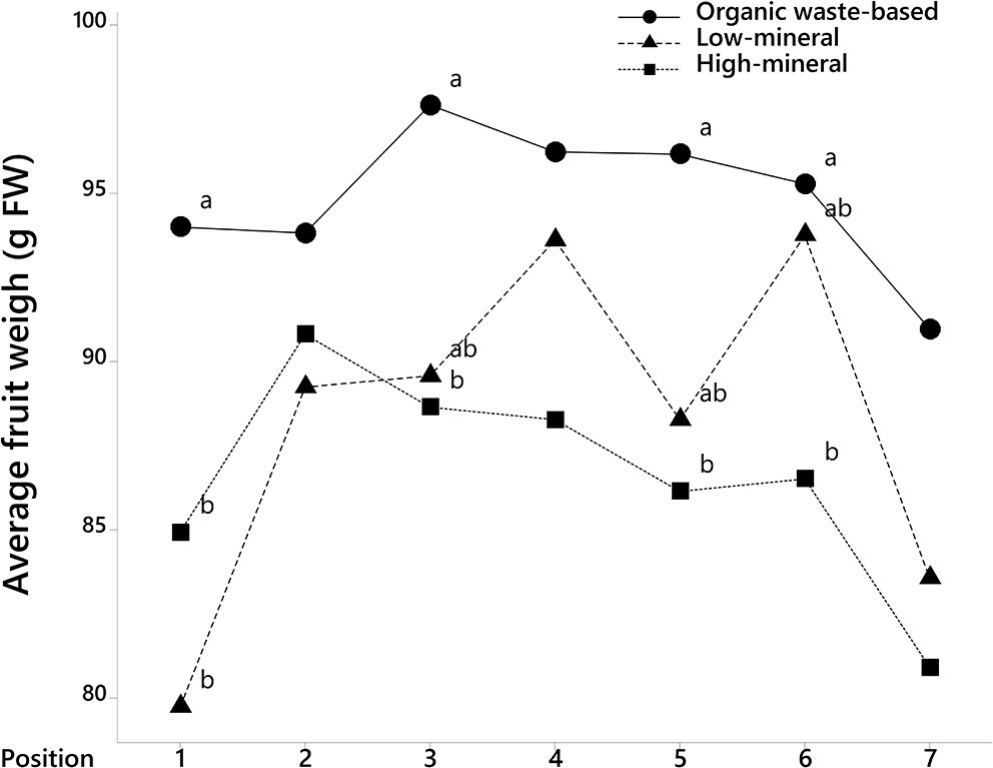
The average weight of tomato fruits at different positions on truss, where 1 is the first to bear fruits on the truss. Mean values are shown. Different letters indicate statistically significant differences at *p* = 0.05, *n* = 47–70.

**Figure 3:**
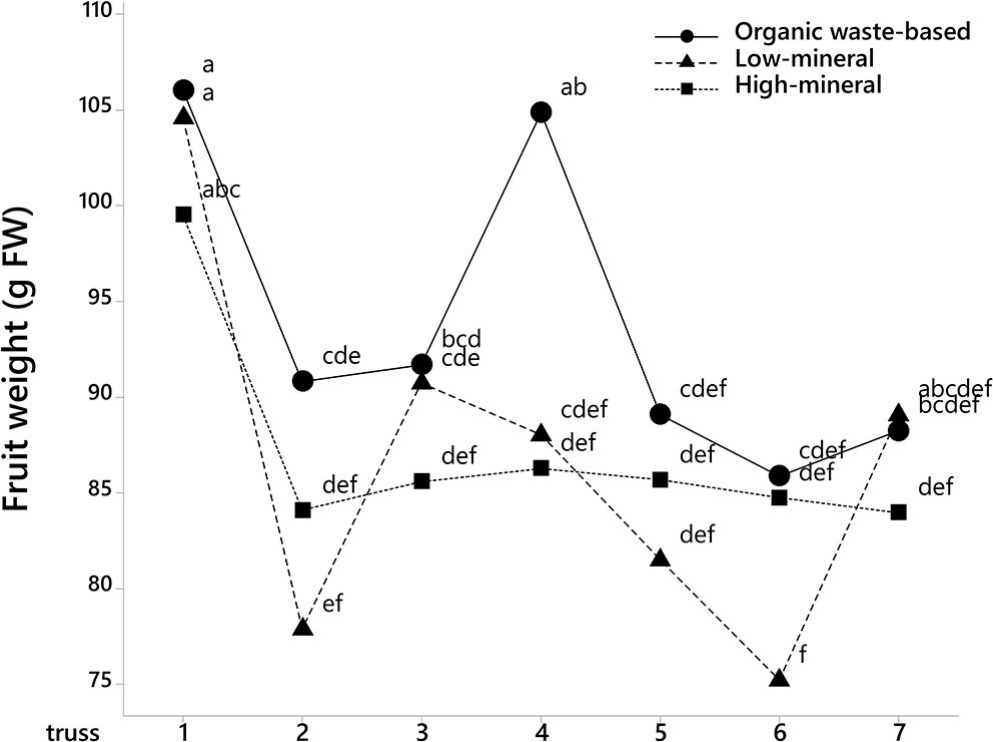
Average tomato fruit weight depends on truss position and treatment, where 1 is the first to develop truss. Mean values are shown. Different letters indicate statistically significant differences at *p* = 0.05, *n* = 23–69.

The analysis of fruit weights with fruit position in the trusses also showed that tomatoes at positions 1, 3, 5, and 6 were significantly larger in the organic waste-based treatment than in the high-mineral treatment (Figure 2). The absence of a significant interaction between treatment and the cluster position of the fruits indicate that the organic waste-based fertilizer did not alter the competition between basal and apical tomatoes in a truss. The fruit weight for the low-mineral treatment was close to that of the high-mineral treatment. However, at positions 4 and 6, the fruit size tended to be higher for the low-mineral than for the high-mineral treatment. We also analyzed tomato weight distribution among trusses and found a significant effect of treatment on fruit size (Figure 3).

### Tomato Plant Biomass and Dry Matter Partitioning

Tomato plants that received organic waste-based nutrition showed the lowest total dry biomass. However, the difference in plant biomass between the organic waste-based and low-mineral treatments was not statistically significant. The different treatments also altered dry matter distribution, with organic waste-based cultivation preferentially stimulating dry matter partitioning to the roots (Table 3). This trend was not observed for the low-mineral treatment, indicating that the greater allocation to the roots was not a consequence of low-mineral nutrient concentrations. Dry matter allocation to the leaves and fruits was similar between the organic waste-based and high-mineral treatments. However, the low-mineral treatment stimulated greater distribution of dry matter to the leaves and a smaller distribution to the fruits. These differences indicate that low concentrations of nutrients diminish dry matter allocation to generative organs. However, the organic waste-based treatment, despite low level of essential nutrients, restored the dry matter distribution from vegetative to generative organs (Table 3).

**Table 3:** Biomass parameters of tomato grown under different nutrient conditions.

**Fertilizer**	**Organic waste-based**	**Low-mineral**	**High-mineral**
Mean total biomass weight (g DW) *n =* 5	1,126 ± 147^a^	1,251 ± 140^a^	1,695 ± 154^b^
Mean root weight (g DW) *n =* 5	40.8 ± 7.4^a^	36.6 ± 4.6^a^	46.7 ± 10.6^a^
DMC of roots (%) *n =* 5	5.6 ± 0.9^a^	4.2 ± 0.3^b^	4.6 ± 0.8^ab^
Partition coefficients *n =* 5 Leaves	30.9 ± 4.4^a^	36.1 ± 2.3^b^	32.1 ± 1.8^ab^
Stems	16.3 ± 1.6^a^	17 ± 1.0^a^	17.2 ± 0.7^a^
Roots	3.6 ± 0.3^a^	2.9 ± 0.3^b^	2.7 ± 0.5^b^
Fruits	49.2 ± 5.7^a^	43.9 ± 3.4^a^	47.9 ± 2.6^a^
Number of leaves at harvest (leaves·plant^−1^) *n =* 10	28.9 ± 3.0^a^	31.6 ± 2.8^a^	36.3 ± 2.1^b^
SLA (cm^2^·g^−1^ DW) *n =* 5	335.4 ± 45.4^a^	238.4 ± 16.8^b^	309.3 ± 20.6^a^
DMC of leaves (g·100 g^−1^ DW) *n =* 10	9.1 ± 0.5^a^	12.6 ± 0.5^b^	10.6 ± 0.3^c^
Plant length (cm) *n =* 10	381 ± 20^ab^	360 ± 16^a^	396 ± 42^b^
Xylem sap exudation rate (mL·h^−1^) *n =* 4	6.5 ± 3.2^a^	13.0 ± 1.5^b^	5.2 ± 1.2^a^

*Mean values ± sd are shown. Different letters indicate statistically significant differences between treatments at p = 0.05*.

The number of leaves correlated with the total biomass values for all treatments, with the highest value observed in the high-mineral treatment and the lowest in the organic waste-based treatment. Specific leaf area (SLA) did not show significant differences between the organic waste-based and high-mineral treatments. However, the SLA was significantly higher for the organic waste-based than for the low-mineral treatment, indicating that low nutrient supply decreased the efficiency of leaf area expansion per unit leaf dry matter, whereas the organic waste-based treatment recovered this trait. The differences in SLA were related to the dry matter percentage in the leaves, indicating that modulation of SLA was not due to differences in leaf thickness but to differences in dry matter content. The plant height and xylem sap flow rates were also more similar between the organic waste-based and high-mineral treatments than for the low-mineral treatment. Taken together, these data indicated that although the plant biomass was significantly lower in the organic waste-based than in the high-mineral treatment, many other plant traits were more phenotypically similar between these two treatments than in plants grown with the low-mineral treatment.

### Fruit Quality

The basic parameters of tomato fruit quality were related to nutrient concentrations in the nutrient solution (Table 4): both the low-mineral and organic waste-based treatments showed significantly lower SSC (15 and 18% lower, respectively), TTA (18 and 20% lower, respectively), and DMC of red fruits (20%). This indicates that low nutrient concentrations in the growth solution directly affect the quality traits of tomato fruits. The tomatoes had 4% lower average firmness when grown with the organic waste-based fertilizer (Table 4) than those grown by using the high-mineral or low-mineral fertilization; this probably reflected the larger fruit size (Table 2).

**Table 4:** Average dry matter content, soluble solids content, total titratable acidity measured in fruits from plants grown under different nutrient conditions, *n* = 9.

**Fertilizer**	**SSC (^**°**^Brix)**	**TTA (CAE)**	**DMC (%)**	**Fruit DW (g)**	**Firmness (Durofel units)**	**pH**
Organic waste-based	4.1 ± 0.1^a^	0.40 ± 0.01^a^	5.2 ± 0.2^a^	4.9 ± 0.7^a^	0.86 ± 0.02^a^	4.7 ± 0.4^a^
Low-mineral	4.2 ± 0.1^a^	0.38 ± 0.03^a^	5.3 ± 0.1^a^	4.5 ± 0.4^a^	0.89 ± 0.02^b^	4.8 ± 0.4^a^
High-mineral	5.0 ± 0.1^b^	0.51 ± 0.03^b^	6.5 ± 0.2^b^	5.6 ± 0.6^b^	0.90 ± 0.01^b^	4.7 ± 0.4^a^

*SSC, soluble solid content; TTA, total titratable acidity; DMC, dry matter content. Mean values ± sd are shown. Different letters indicate statistically significant differences between treatments at p = 0.05*.

**Figure 4:**
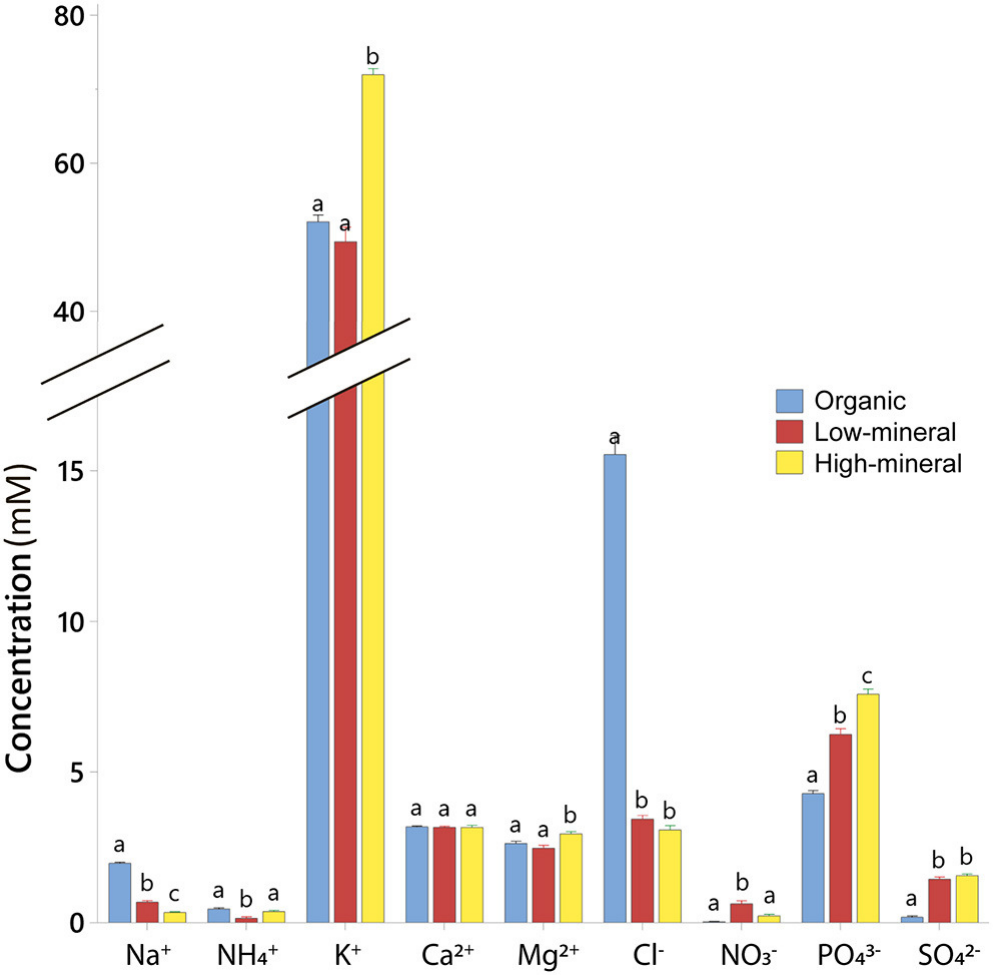
Ionic composition of tomato fruits harvested from plants cultivated with different fertilizers. Mean values ± SD are shown. Different letters indicate statistically significant differences at *p* = 0.05, *n* = 9.

The analysis of fruit ionic composition showed that plants accumulated higher concentrations of Cl^−^ and Na^+^ ions when grown with the organic waste-based treatment compared with the other two treatments (Figure 4, Supplementary Table 2). The tomato fruits grown by organic waste-based fertilization had an ~5-fold higher concentration of Cl^−^ compared with the other treatments, but they showed a near absence of ${\text{NO}}_{3}^{-}$ and the lowest concentrations of ${\text{PO}}_{4}^{3-}$, and ${\text{SO}}_{4}^{2-}$ (57 and 12%, respectively, of the fruit content in the high-mineral treatment). The proportion of inorganic anions to total inorganic ions was significantly lower in the tomato fruits from the high-mineral treatment (36%) than from the organic waste-based or low-mineral treatments (45 and 44%, respectively). This could be linked to the higher accumulation of organic acids in the high-mineral treatment.

### Chemical Composition of Leaves

To characterize leaf function, leaves at the bottom of the canopy were analyzed for their quantities of soluble inorganic ions, pigments, major carbohydrates, and N (Table 5). The leaves showed large differences in ionic composition in the organic waste-based treatment compared with the other two treatments, as the concentration of Na^+^ and Cl^−^ were 7 and 76-fold higher, respectively, in plants from the organic waste-based treatment than plants from the high-mineral treatment. By contrast, ${\text{PO}}_{4}^{3-}$ and ${\text{SO}}_{4}^{-2}$ concentrations were the lowest for the organic waste-based treatments at 21 and 5%, respectively, of the levels in leaves from the high-mineral treatment. These low concentrations indicate that ${\text{PO}}_{4}^{3-}$ and ${\text{SO}}_{4}^{-2}$ can be limiting nutrients in leaves of organic waste-based cultivated plants. Interestingly, the highest concentration of ${\text{NO}}_{3}^{-}$ was found in the leaves of organic waste-based cultivated plants, indicating that deficiency of other nutrients can inhibit ${\text{NO}}_{3}^{-}$ assimilation and cause ${\text{NO}}_{3}^{-}$ accumulation in leaves. The concentration of K^+^ was lower in the organic waste-based and low-mineral treatments than in the high-mineral treatment, confirming that the K^+^ concentration in the nutrient solution defines the ultimate concentration of K^+^ in the leaves. The leaves of organic waste-based cultivated plants were also characterized by the lowest concentration of ${\text{NH}}_{4}^{+}$, which might also be related to the reduction in nitrate assimilation to ${\text{NH}}_{4}^{+}$, as indicated by the highest concentration of nitrate in the leaves.

**Table 5:** Ionic and carbohydrate composition of tomato leaves grown under different nutrient conditions. Chla (b), Chlorophyll a (b); Car, carotenoids.

**Component**	**Organic waste-based**	**Low-mineral**	**High-mineral**
**Inorganic ions (mmol·kg**^**−1**^ **DW)** ***n=*** **10**			
Na^+^	125 ± 37^a^	18 ± 9^b^	40 ± 43^b^
${\text{NH}}_{4}^{+}$	0.4 ± 0.8^a^	2 ± 1.7^b^	2.1 ± 1.5^b^
K^+^	217 ± 59^a^	202 ± 27^a^	341 ± 61^b^
Ca^2+^	143 ± 46^a^	106 ± 22^ab^	84 ± 34^b^
Mg^2+^	192 ± 43^ab^	201 ± 22^a^	152 ± 32^b^
Cl^−^	515 ± 139^a^	7 ± 2^b^	6 ± 2^b^
${\text{NO}}_{3}^{-}$	272 ± 100^a^	66 ± 30^b^	132 ± 50^b^
${\text{PO}}_{4}^{3-}$	21 ± 9^a^	78 ± 11^b^	99 ± 28^c^
${\text{SO}}_{4}^{2-}$	8 ± 8^a^	162 ± 37^b^	150 ± 37^b^
**Pigments (mg·g**^**−1**^ **DW)** ***n=*** **10**			
Chla	12.9 ± 1.0^a^	11 ± 0.7^b^	13.7 ± 1.4^a^
Chlb	3.7 ± 0.4^a^	3.2 ± 0.1^b^	4.0 ± 0.4^a^
Chla+Chlb	16.6 ± 1.3^a^	14.2 ± 0.7^b^	17.6 ± 1.7^a^
Chla/Chlb	3.2 ± 0.4^a^	2.6 ± 0.3^b^	3.3 ± 0.3^a^
Car (x+c)	3.5 ± 0.2^a^	3.4 ± 0.2^a^	3.5 ± 0.1^a^
(Chla+Chlb)/Car	5.2 ± 0.5^a^	5.6 ± 0.6^b^	5.4 ± 0.2^ab^
**Nitrogen content (g·100 g**^**−1**^ **DW)** ***n=*** **10**			
N-${\text{NH}}_{4}^{+}$	0.0007 ± 0.0004^a^	0.0003 ± 0.0004^b^	0.0003 ± 0.0006^b^
N-${\text{NO}}_{3}^{-}$	0.38 ± 0.14^a^	0.09 ± 0.04^b^	0.18 ± 0.07^b^
Organic Nitrogen	2.4 ± 0.2^a^	2.1 ± 0.1^ab^	2.5 ± 0.2^b^
Total Nitrogen	2.8 ± 0.3^a^	2.2 ± 0.1^b^	2.7 ± 0.3^a^
**Carbohydrates (mg·g**^**−1**^ **DW)** ***n=*** **8–10**			
Glucose	9.6 ± 2.5^a^	5.0 ± 1.7^b^	6.0 ± 2.2^b^
Fructose	4.8 ± 1.0^a^	2.8 ± 0.6^b^	4.2 ± 1.1^a^
Sucrose	9.7 ± 1.1^a^	6.8 ± 1.6^b^	7.7 ± 1.0^b^
Starch	24.8 ± 12.4^a^	8.8 ± 6.1^b^	5.0 ± 1.2^b^
Total carbohydrates	46.2 ± 13.5^a^	23.5 ± 7.9^b^	22.9 ± 3.6^b^

*Mean values ± sd are shown. Different letters indicate statistically significant differences between treatments at p = 0.05*.

Plants in the low-mineral treatment had significantly lower leaf contents of chlorophylls a and b compared with the other two treatments (Table 5), although all the three treatments showed the same content of carotenoids (~3.5 mg·g^−1^ DW).

Leaves from the organic waste-based and high-mineral treatments had similar total N content, which was significantly higher than the N content in leaves from the low-mineral treatment. The organic N was, therefore, a major contributor to the observed differences in total N content. The organic waste-based and low-mineral treatments showed similar total N distribution to leaves (73.5 and 73 g N, correspondingly) and fruits (56 and 55 g N, correspondingly). The total N distribution in the high-mineral treatment was 113 g N to leaves and 81 g N to fruits (Supplementary Figure 3).

The leaves of plants grown with the organic waste-based fertilizer had significantly larger concentrations of all analyzed carbohydrates compared with the other two treatments (Table 5). The only exception was a similar concentration of fructose between the leaves from the organic waste-based and high-mineral treatments (4.8 ± 1 and 4.2 ± 1.4 mg·g^−1^ DW, respectively). Glucose, fructose, sucrose, and starch concentrations were 93, 68, 42, and 182% higher in leaves from the organic waste-based treatment than from the low-mineral treatment. The higher accumulation of carbohydrates in leaves from the organic waste-based treatment also coincided with the lowest total biomass in the whole plants, indicating that the reduction of biomass accumulation might arise due to negative feedback inhibition of photosynthesis and accumulation of carbohydrates in the leaves.

The analysis of the carbohydrate content of leaf samples taken at the end of the light period (EL) and the end of the dark period (ED) showed that only glucose concentrations were significantly different between these time periods in leaves from the organic waste-based and high-mineral treatments (Supplementary Figure 2).

### Ionic and Metabolic Composition of Xylem Sap

**Figure 5:**
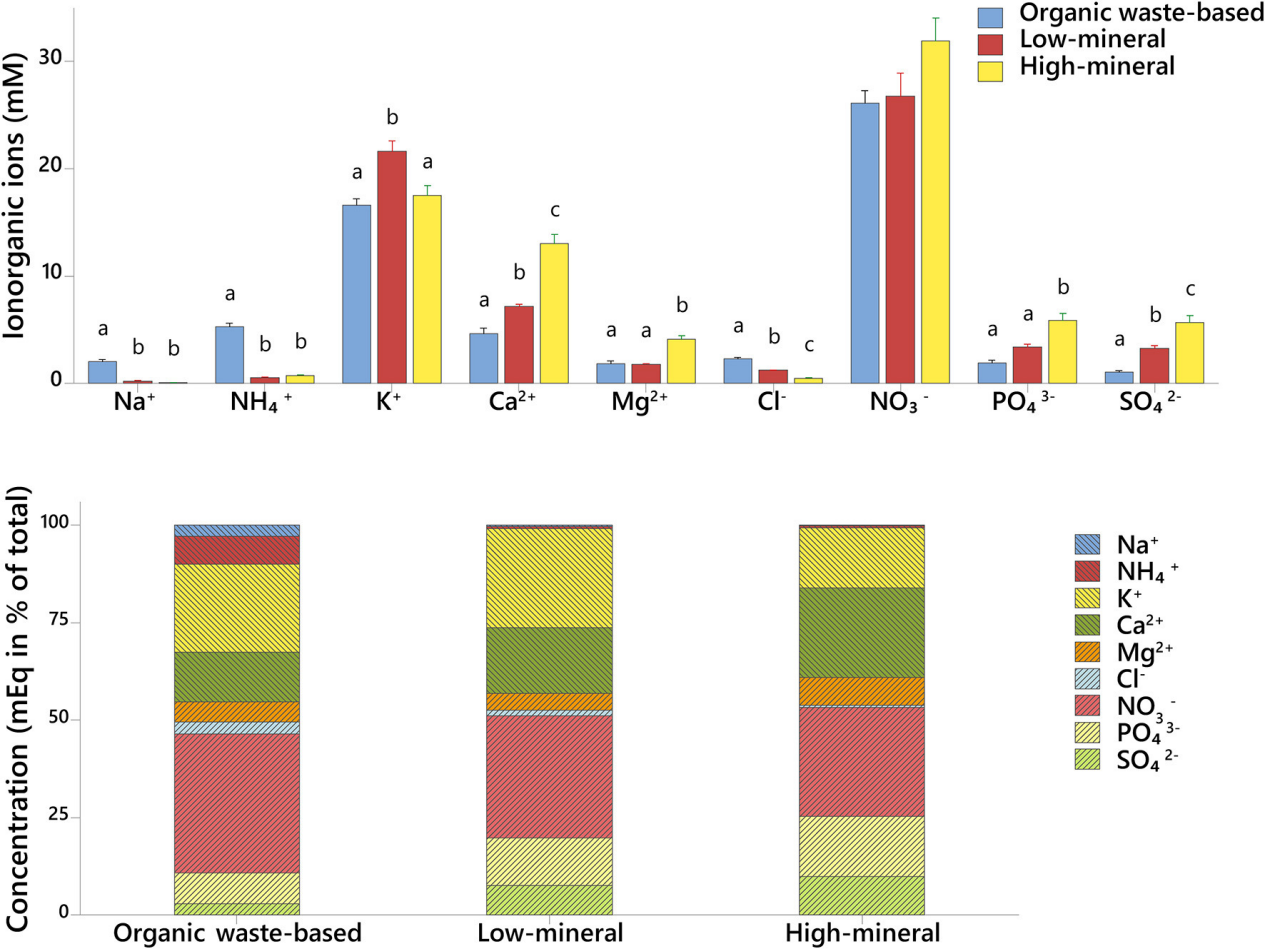
Ionic composition of xylem sap of plants cultivated with different fertilizers. Upper graph, concentration of individual inorganic ions in mmol. Lower graph, ratios of individual inorganic ions in mEq. Mean values ± SD are shown. Different letters indicate statistically significant differences at *p* = 0.05, *n* = 4.

The xylem sap of tomato plants grown with organic waste-based fertilizer had the highest concentration of ${\text{NH}}_{4}^{+}$, Cl^−^, and Na^+^ ions, and exceeded the concentrations found in the high-mineral treatment by 6.6-, 4.6-, and 20-fold, respectively (Figure 5, Supplementary Table 3). The xylem sap of plants from the organic waste-based treatment also had the lowest concentrations of ${\text{SO}}_{4}^{2-}$ and ${\text{PO}}_{4}^{3-}$ among all treatments, 5.4 and 3-fold lower, respectively, than in the high-mineral treatment. ${\text{NO}}_{3}^{-}$ and K^+^ were dominant in all the treatments, and their concentration was not significantly different between the organic waste-based and low-mineral treatments.

**Figure 6:**
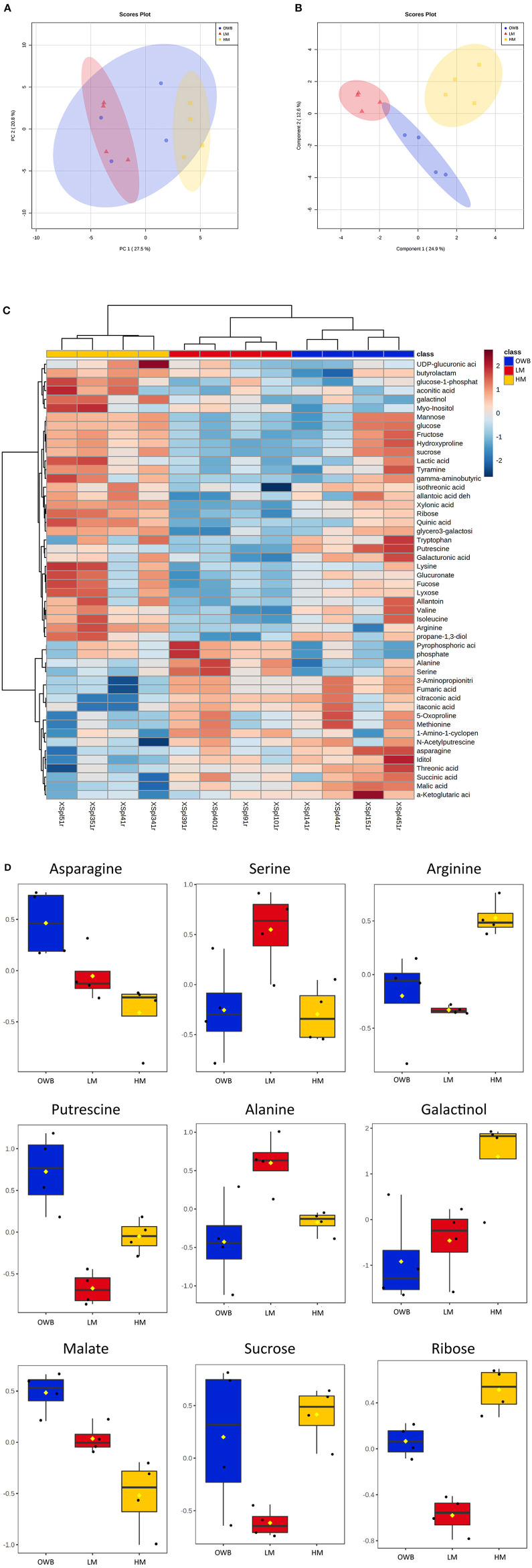
Selected metabolites from the xylem sap of plants cultivated with different fertilizers. Identification is performed in MSDIAL, statistics performed with MetaboAnalyst 5.0 (https://www.metaboanalyst.ca/). Concentrations are shown as log-transformed and normalized mean values. **(A,B)**: Unsupervised and supervised analysis of xylem sap composition, where **(A)** is principal component analysis (PCA) loading plot and **(B)** is sPLS-DA loading plot. **(C)**: Heatmap of 50 most significant metabolites based on ANOVA test. **(D)**: Fold change in selected metabolites for three treatments, *n* = 4. OWB, Organic waste-based; LM, Low-mineral; HM, High-mineral treatments.

Untargeted metabolic analysis was performed with GC-MS on the xylem sap from the three treatments (Figure 6, Supplementary Table 4). An unsupervised multivariate analysis (PCA, Figure 6A) showed significant differences in the xylem sap from the low-mineral and high-mineral treatments, while the xylem sap from the organic waste-based treatment shared a high degree of similarity with both of the other treatments. the supervised analysis clearly separated the treatments (sPLS-DA, Figure 6B) similar to the separation achieved with a heatmap plot, which showed that treatments were correctly separated based on the 50 most significant features according to ANOVA testing (Figure 6C). The organic waste-based treatment had a compositional heterogeneity that was correlated with the cultivation conditions (i.e., samples from plants grown in the same box have similar compositions). Among the significantly different features, the plants in the organic waste-based treatment had the highest concentration of amino compounds (e.g., asparagine and putrescine) and of some organic acids (e.g., malate) (Figure 6D). The high-mineral treatment had the highest concentrations of several compounds, especially those involved in galactose metabolism (e.g., ribose and galactinol) (Figure 6D). The low-mineral treatment generally had the lowest concentrations of the analyzed compounds, such as sucrose (Figure 6D). However, the levels of some amino acids (e.g., serine and alanine) were significantly higher than in the other treatments (Figure 6D).

## Discussion

Tomato cultivation in a closed re-circulating hydroponics system using organic waste-based fertilizer and an integrated nitrification bioreactor was confirmed in this study to produce yields comparable with those achieved with current hydroponic technology. Moreover, we also identified the principal factors that drive the formation of the major components of yield and that modulate fruit quality under organic waste-based plant cultivation. Specifically, we found that (I) organically cultivated plants had a lower growth rate compared to plants cultivated with conventional nutrition, and this was related to the low concentrations of essential nutrient elements in the organic waste-based nutrient solution; (II) fruits of organically cultivated plants were larger in size, that was related to greater availability of Cl^−^ in the nutrient solution of organic hydroponics and subsequently higher Cl^−^ accumulation in the fruits; (III) tomato plants grown with an organic waste-based nutrient solution accumulated more carbohydrate in the leaves; and (IV) fruits cultivated with organic waste-based fertilizer or a low-mineral treatment had lower quality than fruits cultivated with the high-mineral nutrient solution. However, different mechanisms were responsible for the reductions in fruit quality in response to organic waste-based and low-mineral treatments.

### Hydroponics With Organic Waste-Based Fertilizer Decreases Plant Growth, Development, and Yield Because of the Low Concentration of Essential Mineral Elements in the Nutrient Solution

The classical nutrient solution used in industrial hydroponics contains high concentrations of nutrients, exceeding 10 mM for macronutrients like nitrate and potassium (Jones, 2007; Sonneveld and Voogt, 2009), which is the concentration that corresponds to the saturation of the second system of nutrient uptake (George et al., 2012). However, these high concentrations are not necessary for hydroponically grown plants, at least during the early growth stage when plant growth is exponential (Ingestad and Lund, 1986). Numerous experiments examining unlimited, optimal, and suboptimal supplies of nutrients have shown that growth reduction under low concentrations of nutrients in hydroponic solution does not occur only because the concentration is too low for sufficient nutrient uptake but also because of the restricted volume of the nutrient solution: low concentrations result in more rapid and complete depletion of nutrients that occurs with the solution with higher concentrations. Thus, the control of nutrient supply by external concentration is considered to be inadequate because it offers two options for concentration-controlled culture: excess supply or uncontrolled deficiency (Macduff et al., 1993). Taking into account the importance of excluding the complete depletion of nutrients from the solution, we used a large-sized 550 L reservoir and conducted regular monitoring of the concentrations of essential elements in the solution. These approaches allowed us to maintain the continuous presence of essential nutrient elements in solution throughout the entire plant cultivation period in both the organic waste-based and the low-mineral treatments. The fact that plant development (based on the number of trusses, Table 2) decreased in the organic waste-based and low-mineral treatments (despite the verified continuous presence of essential elements in nutrient solution, Table 1) indicates that tomato plants, throughout long-term cultivation, require high concentrations of nutrients typically found in the high-mineral solution used in industrial hydroponics.

Low nutrient concentrations are typical in close-circulation systems, where the main source of essential mineral elements is the digestion of organic fertilizers (Bittsanszky et al., 2016). This type of closed-circulation system is represented by aquaponics, which combines hydroponics and fish production, using the fish effluent, after digestion in a bioreactor, as a source of plant nutrients. In agreement with our data, the low concentration of nutrients in aquaponics solutions is also considered a major challenge that hinders the sustainability of aquaponics as a mode of plant production (Yep and Zheng, 2019). These low concentrations of essential nutrients are less of a problem for leafy vegetables, such as lettuce (Pantanella et al., 2012; Delaide et al., 2016), but can restrict the yield potential for larger plants, such as tomato, which have a longer production season. Tomato productivity was similar to mineral one when using organic fertilizer in soil (Mitchell et al., 2007; Antonious et al., 2019) and short-term hydroponic cultivation (Shinohara et al., 2011). However, only this study compares tomato productivity in soilless hydroponics in long-term cultivation.

Interestingly, the response of tomato plants may not always be explained by differences in mineral composition of nutrient solution. For example, Knaus and Palm (2017) found that the use of tilapia effluent gave higher tomato yield when compared to common carp effluent. The reason for this difference is not clear, but we can assume that the presence of specific biostimulants and/or enrichment of the population of plant growth-promoting bacteria could play a role in this difference. Overall, however, the suboptimal nutrient concentrations in organic hydroponics systems appear to be a major limitation for growing crops that have high nutrient requirements.

Both the organic waste-based and the low-mineral treatments decreased plant growth (Table 3). However, the reason for the growth decrease was not identical, because many agronomic and physiological plant traits showed different responses depending on the nutrition supply. Moreover, the values of many plant traits were more similar to traits of high-mineral plants when the plants were grown in the organic waste-based treatment than when grown in the low-mineral treatment. Specifically, plants cultivated with organic waste-based fertilizer were similar to the high-mineral plants in such traits as distribution of dry matter to fruits and leaves, SLA, plant height, and xylem sap exudation rate from decapitated plants. By contrast, the plants subjected to the low-mineral treatment differed significantly in these traits when compared with either the organically grown or the high-mineral cultivated plants (Table 3). The metabolomic analysis of the xylem sap also showed that metabolite composition under organic waste-based cultivation tended to fall between the high-mineral and low-mineral treatments (Figure 6), further supporting the observations on morphological traits that some as yet unspecified factor available in the organic waste-based system was able to trigger a partial recovery from the “negative” effects of low concentrations of essential elements. However, these factors were not able to recover plant growth in general, as the plants grown with organic waste-based fertilizer had the smallest plant biomass (Table 3).

The low biomass in the organic waste-based treatment might be related to the imbalance of plant nutrition in the organically cultivated plants because the functional leaves of these plants showed very low phosphate and sulfate concentrations (Table 5). The fact that these leaves also accumulated excess carbohydrates supports the assumption that phosphate and/or sulfate deficiency might inhibit assimilate export from the leaves and potentially induce a negative feedback inhibition of photosynthesis under these deficiency conditions. Indeed, phosphate deficiency induces an accumulation of non-structural carbohydrates in leaves (Hammond and White, 2008) that ultimately can decrease photosynthesis and plant growth. Furthermore, the observation that the plants cultivated on organic waste-based fertilizer allocated more dry matter to the roots than plants cultivated in the high-mineral and low-mineral treatments supports the hypothesis that organically cultivated plants experienced nutrient deficiency (Table 3). The trait of dry matter allocation to the roots is very sensitive to nutrient deficiency, as confirmed by numerous investigations (Paponov et al., 1999; Kang and van Iersel, 2004). However, the increased dry matter allocation to the roots in the plants cultivated with organic waste-based fertilizer might also be related to other factors, such as the presence of specific organic compounds. Indeed, stimulatory effects on root growth by organic wastes have been observed in soil-grown citrus trees (Martinez-Alcantara et al., 2016), maize seedlings (Canellas et al., 2002; Jindo et al., 2012), and lettuce in hydroponic culture (Shinohara et al., 2011). Some authors, for example, Compant et al. (2005) and Chinta et al. (2015), have associated this with enhanced root hair formation due to auxin and ethylene produced by microorganisms, whereas Baldi et al. (2010) have linked it to the auxin-like properties of certain humic acids present in organic waste. In addition, humic acids also possess chelating properties that enhance the availability of certain elements, like Fe or Zn. Humic acids, microorganisms, and root hairs are all interconnected, as the initial enhancement of root hair formation improves microbial biofilm development, which further increases root hair growth because of the hormone production by the microorganisms.

Another reason for lower biomass accumulation in organic waste-based treatment may be the presence of organic waste phytotoxins. Plants grown by organic fertilization often develop stress symptoms related to the presence of phytotoxins in organic fertilizer or the accumulation of phytotoxins released by plants. For instance, bok choi plants showed reduced biomass accumulation when cultivated in a closed hydroponic system with plant-derived digestate as a fertilizer (Pelayo Lind et al., 2020).

Oxygenation was provided to both treatments with low EC to improve the nitrification ability of a bioreactor (Ebeling and Timmons, 2012). Saturation of nutrient solution with oxygen was reported to reduce growth self-inhibition of roses in re-circulating cultivation systems (van Os et al., 2012).

### Regulation of Fruit Size Under Organic Waste-Based Production

One interesting observation in this study was that the fruits from organic treatments were larger in size than the fruits from the high-mineral and low-mineral treatments (Table 2). Tomato fruit size is strongly dependent on water flux from the phloem and xylem, because mature tomato fruits have a water content of 92–95% (Guichard et al., 2001), and the extent of water contribution from the phloem and xylem to the fruits depends on plant nutrition. Indeed, reduction in the nutrient concentrations in the low-mineral treatment strongly increased root power (based on the xylem sap rate of decapitated plants, Table 3), indicating a higher contribution from water moving through the xylem. This observation is in agreement with numerous other observations of the effects of nutrient concentrations on xylem sap flow rate (Ehret and Ho, 1986). Interestingly, the fruit size for the low-mineral treatment was not significantly increased beyond that seen in high-mineral nutrition, suggesting that the higher xylem flux to the fruits was compensated by a lower flux from the phloem, resulting in unchanged final fruit size.

Surprisingly, plants cultivated with the organic waste-based fertilizer, despite the similarities in the ion concentrations in the nutrient solution to those in the low-mineral treatment, had a xylem sap flow rate half that observed in the low-mineral plants and was more similar to the xylem sap flow rate in the high-mineral solution. This indicates that some other factor specific to the plants cultivated with organic waste-based fertilizer was offsetting the positive effect of low ion concentrations on root power. The root power, and therefore the water flux through the xylem, was not enhanced in the plants cultivated with organic waste-based nutrition compared with the plants in the high-mineral nutrition, and yet the size of the fruits was significantly larger in the plants cultivated with organic waste-based fertilizer. This suggests that some alternative form of regulation of fruit size is occurring under the organic nutrition conditions, independent of root power regulation.

Detailed ionic and metabolomics analysis, as well as determination of the xylem sap rate of decapitated plants, strongly pointed to a potential role of Cl^−^ in the fruit size increase in the plants growing in organic hydroponics. Indeed, fruit analysis showed about a 5-fold higher Cl^−^ accumulation in the plants cultivated in the organic waste-based treatment than in the plants cultivated in the low-mineral and high-mineral treatments (Figure 4). High salinity is often responsible for reductions in fruit size (Oliveira et al., 2013; Zhang et al., 2016), but at intermediate concentrations, the replacement of ${\text{NO}}_{3}^{-}$ with Cl^−^ causes no effects of Cl^−^ on fruit size (Chapagain et al., 2003). However, the effect of low chloride concentrations on the nutrient solutions with low electrical conductivity (EC) has not been investigated before. A recent investigation of the role of Cl^−^ in shoot growth showed that under non-saline conditions (up to 5 mM Cl^−^) and with no water limitations, Cl^−^ specifically stimulated an increase in leaf cell size and led to a moderate increase in biomass because of greater cell expansion (Franco-Navarro et al., 2015). Application of Cl^−^ in a 1–5 mM range indicated specific roles for Cl^−^ in regulating leaf osmotic potential and turgor. Clearly, these effects of Cl^−^, if also occurring in tomato fruits, could reduce the fruit quality of tomatoes produced with organic waste-based fertilizer.

### Quality of Tomato Fruits Under Organic Waste-Based Production

Organically cultivated fruits are generally perceived to have higher quality than conventionally grown fruits. In this experiment, tomatoes cultivated in hydroponics with organic waste-based fertilizer had lower fruit quality, based on common characteristics such as SSC, TTA, and DMC (Table 2). Accumulation of soluble carbohydrates, which are the main component of tomato fruit quality, is strongly dependent on the contribution of phloem and xylem fluxes to the fruits. Direct comparison of the two mineral treatments (high-mineral vs. low-mineral) showed that an increased contribution of xylem flux over phloem flux due to higher root power in the low-mineral treatment (because of increased xylem sap rate) might be the main cause of decreased fruit quality because the concentration of sucrose is much higher in the phloem than in the xylem (Nakamura et al., 2008; Jacobs et al., 2012).

The lower quality of fruits of the plants cultivated with the organic waste-based fertilizer, in contrast, appears to arise *via* other mechanisms than increased xylem flux, because the xylem sap flux in decapitated plants was the same as that of organically cultivated and high-mineral plants. The lower quality might be related to a dilution effect that gave rise to the larger fruits. As mentioned before, the higher concentration of Cl^−^ in the nutrient solution, and ultimately in the fruits, might be responsible for the larger fruit size. Moreover, high concentration of Cl^−^ in the fruits may have osmotic effects that can result in reduced demands for the accumulation of low-molecular-weight organic compounds (organic acids and monosaccharides) required to maintain fruit turgor.

The effect of NaCl on fruit quality is strongly dependent on the NaCl concentration in the nutrient solution. High NaCl concentrations (above 100 mM) that cause salt stress typically increase the concentrations of soluble carbohydrates (Zhang et al., 2016) but may also cause blossom end rot in tomatoes because of reduction in the uptake of K, Ca, Mg, and P (Sonneveld and Voogt, 2009; Hagassou et al., 2019). Investigations of moderate NaCl applications (17 mM) have shown no deleterious effects of Cl^−^ on fruit quality (Montesano et al., 2010).

Interestingly, low concentration of soluble carbohydrates was observed in the fruits under organic waste-based cultivation, despite the highest accumulation of non-structural carbohydrates in the leaves with this treatment. This indicates that regulation of the export of carbohydrates from the leaves participates in the regulation of soluble sugar accumulation in fruits.

### Common Recommendations for Organic Waste-Based Fertilizers Used in Hydroponics

In this study, organic waste was used as a base for fertilizer formulation. However, its elemental composition was not sufficient for normal growth of tomato plants over 6 months. Therefore, the liquid digestate was supplemented with essential microelements, magnesium, calcium, sulfate, and phosphate ions. When necessary, the pH was adjusted with nitric acid or potassium hydroxide.

Maintaining an optimal pH (5.5–6.5) of the nutrient solution is very important for nutrient availability in the hydroponic cultivation of tomatoes (Hosseinzadeh et al., 2017). In particular, the pH (7.5–8.5) will reduce the availability of Fe, Mn, and Zn to the plants (Resh, 2012). The average pH of the organic waste-based and high-mineral solutions was below the recommended upper limit (6.3 and 6.4, correspondingly), while the low-mineral solution showed a higher average value of pH 7 (Table 1), which might only partially limit nutrient availability. At the same time, pH values of up to 7 did not affect yield and DMC of cucumber in fertilization systems that incorporated an integrated nitrification bioreactor (Tyson et al., 2008).

Despite constant recirculation of the nutrient solution, the variation in pH level was highest for the organic waste-based fertilizer (SD = 0.99, Table 1). One explanation is the batch addition of the high-pH organic waste (9.7), which temporarily increased the pH of the fertilization solution. With time, as ammonium was nitrified to nitrate, the pH again declined. A large variation in pH was also reported in the cultivation of bok choi in a closed system with an integrated MBBR (Pelayo Lind et al., 2020).

This strong pH variation in the nutrient solution might be responsible for the chlorosis observed in the upper leaves in the canopy of plants grown with organic waste-based fertilizer, assuming this chlorosis was a consequence of deficiency in non-recirculating nutrients. The pH fluctuations after the addition of new organic fertilizer could potentially influence the uptake of microelements (e.g., Fe and Zn, Marschner, 1995), as these microelements have a low reutilization rate from previously accumulated sources in plants, such as old leaves. Consequently, their deficiency appears on the upper, most recently expanded leaves. However, we cannot exclude the potential influence of the organic component of the organic waste-based fertilizer, as this had a high concentration of sterols, lipids, fatty acids, and especially phenolic compounds (data not shown).

Overall, this study showed that fertilizer based on a liquid by-product of biogas production from livestock manure can be used for the hydroponic cultivation of tomatoes in a greenhouse. However, the nutrient content requires optimization by the selective addition of minerals or by the addition of a combination of different organic wastes for optimal plant development. The use of organic waste-based fertilizer is favorable from the circular economy point of view but has several limitations, such as production of lower quality fruit, need for nitrification, and control of the nutrient composition of fertilizer. The use of this fertilizer should therefore be policy-driven or should operate from a niche in the market where it will not compete with tomatoes produced by cultivation with mineral fertilizers.

We have designed and constructed a closed re-circulating cultivation system with an integrated nitrification bioreactor. We have compared plant development, fruit yield, and quality of tomatoes grown with a fertilizer based on organic waste (the liquid by-product of biogas production from pig manure) with greenhouse tomatoes grown with mineral fertilizers. The tomatoes grown with the organic waste-based fertilizer had a similar yield but poorer taste characteristics when compared with tomatoes grown with the high-mineral fertilizer. The plants grown with organic waste-based fertilizer had larger average fruit weight, smaller proportion of leaves, and lower DMC of the leaves compared to plants grown with the low-mineral fertilizer. The plants grown with the organic waste-based treatment accumulated higher amount of salts, especially Cl^−^, in their tissues. Overall, fertilizers based on organic wastes change plant development toward a generative state and can partially recover the physiological and biochemical responses seen in plants grown under suboptimal fertilization conditions, suggesting that these fertilizers could be favored over mineral fertilizers with similar inorganic compositions. However, the use of organic waste-based fertilizers is less feasible than high-mineral fertilizers because of the lower quality of tomato fruits produced.

## Data Availability Statement

The original contributions presented in the study are included in the article/Supplementary Material, further inquiries can be directed to the corresponding author/s.

## Author Contributions

DK, IP, and MV planned and designed the experiment. DK performed the experiment and analysis of samples. MP and AP substantially contributed to the establishment of analytical procedures for chemical analysis of leaves and to sample collection and analysis. DK drafted the manuscript with a substantial contribution from IP, MV, and MP. All authors contributed to the article and approved the submitted version.

## Conflict of Interest

The authors declare that the research was conducted in the absence of any commercial or financial relationships that could be construed as a potential conflict of interest.

## Abbreviations

COD: chemical oxygen demand
DMC: dry matter content
DO: dissolved oxygen
DW: dry weight
EC: electrical conductivity
ED: end of dark
EL: end of light
FW: fresh weight
GC–MS: gas chromatography–mass spectrometry
HK: hexokinase
IQR: interquartile range
MBBR: moving bed bioreactor
MeOH: methanol
MSD: mass selective detector
MSTFA: N-trimethylsilyl-N-methyl trifluoroacetamide
MTBE: methyl *tert*-butyl ether
ORP: oxidation-reduction potential
PCA: principal component analysis
SLA: specific leaf area
sPLS-DA: sparse partial least squares discriminant analysis
SS: soluble solids
SSC: soluble solid content
TS: total solid
TTA: total titratable acidity
VS: volatile solid.

## References

[B1] Alexou M. Peuke A. D. (2013). “Methods for xylem sap collection,” in Plant Mineral Nutrients: Methods and Protocols, ed F. J. M. Maathuis (Totowa, NJ: Humana Press), 195–207. 10.1007/978-1-62703-152-3_13 23073885

[B2] Antonious G. Turley E. Dawood M. (2019). Ascorbic acid, sugars, phenols, and nitrates concentrations in tomato grown in animal manure amended soil. Agriculture 9:94. 10.3390/agriculture9050094

[B3] Baldi E. Toselli M. Eissenstat D. M. Marangoni B. (2010). Organic fertilization leads to increased peach root production and lifespan. Tree Physiol. 30, 1373–1382. 10.1093/treephys/tpq078 20921024

[B4] Bittsanszky A. Uzinger N. Gyulai G. Mathis A. Junge R. Villarroel M.. (2016). Nutrient supply of plants in aquaponic systems. Ecocycles 2, 17–20. 10.19040/ecocycles.v2i2.57

[B5] Canellas L. P. Olivares F. L. Okorokova-Facanha A. L. Facanha R. A. (2002). Humic acids isolated from earthworm compost enhance root elongation, lateral root emergence, and plasma membrane h+-atpase activity in maize roots. Plant Physiol. 130, 1951–1957. 10.1104/pp.007088 12481077 PMC166705

[B6] Carpenter S. R. Caraco N. F. Correll D. L. Howarth R. W. Sharpley A. N. Smith H. V. (1998). Nonpoint pollution of surface waters with phosphorus and nitrogen. Ecol. Appl. 8, 559–568. 10.1890/1051-0761(1998)008[0559:NPOSWW]2.0.CO;2

[B7] Chapagain B. P. Wiesman Z. Zaccai M. Imas P. Magen H. (2003). Potassium chloride enhances fruit appearance and improves quality of fertigated greenhouse tomato as compared to potassium nitrate. J. Plant Nutr. 26, 643–658. 10.1081/PLN-120017671

[B8] Cheng J. Shearin T. E. Peet M. M. Willits D H. (2004). Utilization of treated swine wastewater for greenhouse tomato production. Water Sci. Technol. 50, 77–82. 10.2166/wst.2004.0093 15344776

[B9] Chinta Y. D. Eguchi Y. Widiastuti A. Shinohara M. Sato T. (2015). Organic hydroponics induces systemic resistance against the air-borne pathogen, botrytis cinerea (gray mould). J. Plant Interact. 10, 243–251. 10.1080/17429145.2015.1068959

[B10] Chong J. Wishart D. S. Xia J. (2019). Using metaboanalyst 4.0 for comprehensive and integrative metabolomics data analysis. Curr. Protoc. Bioinform. 68:e86. 10.1002/cpbi.86 31756036

[B11] Chow P. S. Landhausser S. M. (2004). A method for routine measurements of total sugar and starch content in woody plant tissues. Tree Physiol. 24, 1129–1136. 10.1093/treephys/24.10.1129 15294759

[B12] Compant S. Duffy B. Nowak J. Clement C. Barka A. E. (2005). Use of plant growth-promoting bacteria for biocontrol of plant diseases: principles, mechanisms of action, and future prospects. Appl. Environ. Microbiol. 71, 4951–4959. 10.1128/AEM.71.9.4951-4959.2005 16151072 PMC1214602

[B13] De Borba B. M. Jack R. F. Rohrer J. S. Wirt J. Wang D. (2014). Simultaneous determination of total nitrogen and total phosphorus in environmental waters using alkaline persulfate digestion and ion chromatography. J. Chromatogr. A 1369, 131–137. 10.1016/j.chroma.2014.10.027 25441080

[B14] Delaide B. Goddek S. Gott J. Soyeurt H. H Jijakli M. (2016). Lettuce (lactuca sativa l. Var. Sucrine) growth performance in complemented aquaponic solution outperforms hydroponics. Water 8:467. 10.3390/w8100467

[B15] Ebeling J. M. Timmons M. B. (2012). “Recirculating aquaculture systems,” in Aquaculture Production Systems, ed J. H. Tidwell (Ames: John Wiley and Sons), 245–277. 10.1002/9781118250105.ch11

[B16] Ehret D. L. Ho L. C. (1986). Translocation of calcium in relation to tomato fruit growth. Ann. Bot. 58, 679–688. 10.1093/oxfordjournals.aob.a087230

[B17] Ehret D. L. Menzies J. G. Helmer T. (2005). Production and quality of greenhouse roses in recirculating nutrient systems. Sci. Hortic. 106, 103–113. 10.1016/j.scienta.2005.03.002

[B18] FAOSTAT (2019). Livestock Manure 2018. Rome: Statistical Division of the UN Food and Agriculture Organization. Available online at: http://www.fao.org/faostat/en/#data/EMN

[B19] Favoino E. Hogg D. (2008). The potential role of compost in reducing greenhouse gases. Waste Manag. Res. 26, 61–69. 10.1177/0734242X08088584 18338702

[B20] Fiehn O. (2003). Metabolic networks of cucurbita maxima phloem. Phytochemistry 62, 875–886. 10.1016/S0031-9422(02)00715-X 12590115

[B21] Franco-Navarro J. D. Brumós J. Rosales M. A. Cubero-Font P. Talón M. Colmenero-Flores M. J. (2015). Chloride regulates leaf cell size and water relations in tobacco plants. J. Exp. Bot. 67, 873–891. 10.1093/jxb/erv502 26602947 PMC4737079

[B22] George E. Horst W. J. Neumann E. (2012). “Chapter 17 - adaptation of plants to adverse chemical soil conditions,” in Marschner's Mineral Nutrition of Higher Plants (third edition), ed P. Marschner (San Diego, CA: Academic Press), 409–472. 10.1016/B978-0-12-384905-2.00017-0

[B23] Guichard S. Bertin N. Leonardi C. Gary C. (2001). Tomato fruit quality in relation to water and carbon fluxes. Agronomie 21, 385–392. 10.1051/agro:2001131

[B24] Hagassou D. Francia E. Ronga D. Buti M. (2019). Blossom end-rot in tomato (solanum lycopersicum l.): a multi-disciplinary overview of inducing factors and control strategies. Sci. Hortic. 249, 49–58. 10.1016/j.scienta.2019.01.042

[B25] Hammond J. P. White P. J. (2008). Sucrose transport in the phloem: integrating root responses to phosphorus starvation. J. Exp. Bot. 59, 93–109. 10.1093/jxb/erm221 18212031

[B26] Hill C. B. Roessner U. (2013). “Metabolic profiling of plants by GC-MS,” in The Handbook of Plant Metabolomics, eds W. Weckwerth and G. Kahl. Weinheim (Wiley-VCH Verlag GmbH & Co. KGaA), 1–23. 10.1002/9783527669882.ch1

[B27] Hosseinzadeh S. Verheust Y. Bonarrigo G Van Hulle, S. (2017). Closed hydroponic systems: Operational parameters, root exudates occurrence and related water treatment. Rev. Environ. Sci. Bio-Technol. 16, 59–79. 10.1007/s11157-016-9418-6

[B28] Hostettler C. Kölling K. Santelia D. Streb S. Kötting O. Zeeman C. S. (2011). “Analysis of starch metabolism in chloroplasts,” in Chloroplast Research in Arabidopsis: Methods and Protocols, Vol. ii. ed R. P. Jarvis (Totowa, NJ, Humana Press), 387–410. 10.1007/978-1-61779-237-3_21 21863455

[B29] Ingestad T. Lund A. B. (1986). Theory and techniques for steady state mineral nutrition and growth of plants. Scand. J. Forest Res. 1, 439–453. 10.1080/02827588609382436

[B30] Jacobs J. M. Babujee L. Meng F. H. Milling A. Allen C. (2012). The in planta transcriptome of ralstonia solanacearum: conserved physiological and virulence strategies during bacterial wilt of tomato. Mbio 3:e00114–2. 10.1128/mBio.00114-12 22807564 PMC3413399

[B31] Jindo K. Martim S. A. Navarro E. C. Perez-Alfocea F. Hernandez T. Garcia C.. (2012). Root growth promotion by humic acids from composted and non-composted urban organic wastes. Plant Soil 353, 209–220. 10.1007/s11104-011-1024-3

[B32] Jones J. B. Jr. (2007). Tomato Plant Culture. CRC press. 10.1201/9781420007398

[B33] Kang J. G. van Iersel M. W. (2004). Nutrient solution concentration affects shoot: Root ratio, leaf area ratio, and growth of subirrigated salvia (salvia splendens). Hortscience 39, 49–54. 10.21273/HORTSCI.39.1.49

[B34] Knaus U. Palm H. W. (2017). Effects of the fish species choice on vegetables in aquaponics under spring-summer conditions in northern germany (mecklenburg western pomerania). Aquaculture 473, 62–73. 10.1016/j.aquaculture.2017.01.020

[B35] Lai Z. Tsugawa H. Wohlgemuth G. Mehta S. Mueller M. Zheng Y.. (2018). Identifying metabolites by integrating metabolome databases with mass spectrometry cheminformatics. Nat. Methods 15, 53–56. 10.1038/nmeth.4512 29176591 PMC6358022

[B36] Lichtenthaler H. K. Buschmann C. (2001). Chlorophylls and carotenoids: Measurement and characterization by uv-vis spectroscopy. Curr. Protoc. Food Anal. Chem. 1, F4.3.1–F4.3.8. 10.1002/0471142913.faf0403s01

[B37] Liedl B. E. Bombardiere J. Chaffield M. J. (2006). Fertilizer potential of liquid and solid effluent from thermophilic anaerobic digestion of poultry waste. Water Sci. Technol. 53, 69–79. 10.2166/wst.2006.237 16784191

[B38] Macduff J. H. Jarvis S. C. Larsson C.-M. Oscarson P. (1993). Plant growth in relation to the supply and uptake of ${\text{no}}_{3}^{-}$: a comparison between relative addition rate and external concentration as driving variables. J. Exp. Bot. 44, 1475–1484. 10.1093/jxb/44.9.1475

[B39] Maessen H. Verheul M. (2016). Vurdering av avrenningsvann i veksthusgrønnsaker. NIBIO Rapport. 2:28. Available online at: http://hdl.handle.net/11250/2441050

[B40] Magalhaes J. R. Wilcox G. E. (1984). Ammonium toxicity development in tomato plants relative to nitrogen form and light-intensity. J. Plant Nutr. 7, 1477–1496. 10.1080/01904168409363295

[B41] Mallin M. A. Cahoon L. B. (2003). Industrialized animal production - a major source of nutrient and microbial pollution to aquatic ecosystems. Popul. Environ. 24, 369–385. 10.1023/A:1023690824045

[B42] Marschner H. (1995). “15 - the soil–root interface (rhizosphere) in relation to mineral nutrition,” in Marschner's Mineral Nutrition of Higher Plants (second edition), ed H. Marschner (San Diego, CA: Academic Press), 541. 10.1016/B978-012473542-2/50001-8

[B43] Martinez-Alcantara B. Martinez-Cuenca M. R. Bermejo A. Legaz F. Quinones A. (2016). Liquid organic fertilizers for sustainable agriculture: nutrient uptake of organic versus mineral fertilizers in citrus trees. PLoS ONE 11:e0161619. 10.1371/journal.pone.0161619 27764099 PMC5072554

[B44] Mateo-Sagasta J. Zadeh S. M. Turral H. Burke J. (2017). Water Pollution From Agriculture: A Global Review. Executive Summary. Rome, Italy: Food and Agriculture Organization of the United Nations (FAO); Colombo: International Water Management Institute (IWMI).

[B45] Mitcham B. Cantwell M. Kader A. (1996). Methods for determining quality of fresh commodities. Perishables Handling Newsletter 1–5. Available online at: https://ucanr.edu/datastoreFiles/608-295.pdf

[B46] Mitchell A. E. Hong Y. J. Koh E. Barrett D. M. Bryant D. E. Denison R. F.. (2007). Ten-year comparison of the influence of organic and conventional crop management practices on the content of flavonoids in tomatoes. J. Agric. Food Chem. 55, 6154–6159. 10.1021/jf070344+ 17590007

[B47] Möller K. Müller T. (2012). Effects of anaerobic digestion on digestate nutrient availability and crop growth: a review. Eng. Life Sci. 12, 242–257. 10.1002/elsc.201100085

[B48] Montesano F. Parente A. Santamaria P. (2010). Closed cycle subirrigation with low concentration nutrient solution can be used for soilless tomato production in saline conditions. Sci. Hortic. 124, 338–344. 10.1016/j.scienta.2010.01.017

[B49] Nakamura S.- I. Akiyama C. Sasaki T. Hattori H. Chino M. (2008). Effect of cadmium on the chemical composition of xylem exudate from oilseed rape plants (brassica napus l.). Soil Sci. Plant Nutr. 54, 118–127. 10.1111/j.1747-0765.2007.00214.x

[B50] Oliveira A. B. Moura C. F. Gomes-Filho E. Marco C. A. Urban L. Miranda R. M. (2013). The impact of organic farming on quality of tomatoes is associated to increased oxidative stress during fruit development. PLoS ONE 8:e56354. 10.1371/journal.pone.0056354 23437115 PMC3577808

[B51] Pantanella E. Cardarelli M. Colla G. Rea E. Marcucci A. (2012). Aquaponics vs. Hydroponics: Production and Quality of Lettuce Crop. Leuven: International Society for Horticultural Science (ISHS). 10.17660/ActaHortic.2012.927.109

[B52] Paponov I. A. Lebedinskai S. I Koshkin E. (1999). Growth analysis of solution culture-grown winter rye, wheat and triticale at different relative rates of nitrogen supply. Ann. Bot. 84, 467–473. 10.1006/anbo.1999.0935

[B53] Paponov M. Kechasov D. Lacek J. Verheul M. J. Paponov I. A. (2020). Supplemental light-emitting diode inter-lighting increases tomato fruit growth through enhanced photosynthetic light use efficiency and modulated root activity. Front. Plant Sci. 10:1656. 10.3389/fpls.2019.01656 31998343 PMC6965351

[B54] Pelayo Lind O. Hultberg M. Bergstrand K.-J. Larsson-Jönsson H. Caspersen S. Asp H. (2020). Biogas digestate in vegetable hydroponic production: Ph dynamics and ph management by controlled nitrification. Waste Biomass Valoriz. 12, 123–133. 10.1007/s12649-020-00965-y

[B55] Poustkova I. Kourimska L. Vaclavikova K. Miholova D. Babicka L. (2009). The effect of fertilization method on selected elements content in tomatoes (lycopersicon lycopersicum). Czech J. Food Sci. 27, S394–S396. 10.17221/599-CJFS

[B56] Purcell L. C. King C. A. (1996). Total nitrogen determination in plant material by persulfate digestion. Agron. J. 88, 111–113. 10.2134/agronj1996.00021962008800010023x

[B57] Resh H. M. (2012). Hydroponic Food Production: A Definitive Guidebook for the Advanced Home Gardener and the Commercial Hydroponic Grower. Boca Raton, FL: CRC Press. 10.1201/b12500

[B58] Rusten B. Eikebrokk B. Ulgenes Y. Lygren E. (2006). Design and operations of the kaldnes moving bed biofilm reactors. Aquac. Eng. 34, 322–331. 10.1016/j.aquaeng.2005.04.002

[B59] Saijai S. Ando A. Inukai R. Shinohara M. Ogawa J. (2016). Analysis of microbial community and nitrogen transition with enriched nitrifying soil microbes for organic hydroponics. Biosci. Biotechnol. Biochem. 80, 2247–2254. 10.1080/09168451.2016.1200459 27351990

[B60] Salem M. A. Juppner J. Bajdzienko K. Giavalisco P. (2016). Protocol: a fast, comprehensive and reproducible one-step extraction method for the rapid preparation of polar and semi-polar metabolites, lipids, proteins, starch and cell wall polymers from a single sample. Plant Methods 12:45. 10.1186/s13007-016-0146-2 27833650 PMC5103428

[B61] Sarker S. Lamb J. J. Hjelme D. R. Lien M. K. (2019). A review of the role of critical parameters in the design and operation of biogas production plants. Appl. Sci. Basel 9:1915. 10.3390/app9091915

[B62] Shinohara M. Aoyama C. Fujiwara K. Watanabe A. Ohmori H. Uehara Y.. (2011). Microbial mineralization of organic nitrogen into nitrate to allow the use of organic fertilizer in hydroponics. Soil Sci. Plant Nutr. 57, 190–203. 10.1080/00380768.2011.554223

[B63] Sonneveld C. Voogt W. (2009). Plant Nutrition of Greenhouse Crops. Dordrecht: Springer Netherlands. 10.1007/978-90-481-2532-6

[B64] Tsugawa H. Cajka T. Kind T. Ma Y. Higgins B. Ikeda K.. (2015). Ms-dial: Data-independent ms/ms deconvolution for comprehensive metabolome analysis. Nat. Methods 12, 523–526. 10.1038/nmeth.3393 25938372 PMC4449330

[B65] Tyson R. V. Simonne E. H. Treadwell D. D. Davis M. M White J. (2008). Effect of water ph on yield and nutritional status of greenhouse cucumber grown in recirculating hydroponics. J. Plant Nutr. 31, 2018–2030. 10.1080/01904160802405412

[B66] Van Os E. A. (1999). Closed soilless growing systems: a sustainable solution for dutch greenhouse horticulture. Water Sci. Technol. 39, 105–112. 10.2166/wst.1999.0228

[B67] van Os E. A. Van Der Maas A. A. Meijer R. J. M. Khodabaks M. R. Blok C. Enthoven N. L. M. (2012). Advanced oxidation to eliminate growth inhibition and to degrade plant protection products in a recirculating nutrient solution in rose cultivation. Acta Hortic. 927, 941–947. 10.17660/ActaHortic.2012.927.116

[B68] Verheul M. J. (2005). “A rational growing system for organic production of greenhouse tomatoes,” Proceedings NJF-Seminar. 369: Organic Farming for a New Millennium - Status and Future Challanges (Alnarp).

[B69] Verheul M. J. Slimestad R. H Tjostheim I. (2015). From producer to consumer: Greenhouse tomato quality as affected by variety, maturity stage at harvest, transport conditions, and supermarket storage. J. Agric. Food Chem. 63, 5026–5034. 10.1021/jf505450j 25916229

[B70] Warren C. R. (2008). Rapid measurement of chlorophylls with a microplate reader. J. Plant Nutr. 31, 1321–1332. 10.1080/01904160802135092

[B71] Woodward G. Gessner M. O. Giller P. S. Gulis V. Hladyz S. Lecerf A.. (2012). Continental-scale effects of nutrient pollution on stream ecosystem functioning. Science 336, 1438–1440. 10.1126/science.1219534 22700929

[B72] Yep B. Zheng Y. (2019). Aquaponic trends and challenges – a review. J. Clean. Product. 228, 1586–1599. 10.1016/j.jclepro.2019.04.290

[B73] Zhai Z. Ehret D. L. Forge T. Helmer T. Lin W. Dorais M.. (2009). Organic fertilizers for greenhouse tomatoes: productivity and substrate microbiology. HortScience 44, 800–809. 10.21273/HORTSCI.44.3.800

[B74] Zhang P. Senge M. Dai Y. (2016). Effects of salinity stress on growth, yield, fruit quality and water use efficiency of tomato under hydroponics system. Rev. Agric. Sci. 4, 46–55. 10.7831/ras.4.46

[B75] Zhao D. MacKown C. T. Starks P. J. K Kindiger B. (2010). Rapid analysis of nonstructural carbohydrate components in grass forage using microplate enzymatic assays. Crop Sci. 50, 1537–1545. 10.2135/cropsci2009.09.0521

