# Supplementary material for: Organic Waste-Based Fertilizer in Hydroponics Increases Tomato Fruit Size but Reduces Fruit Quality
Source: Front Plant Sci. 2021 Jun 23;12:680030. doi: 10.3389/fpls.2021.680030 (PMC8261069; doi:10.3389/fpls.2021.680030)
Supplement: Supplementary file 1 [file Data_Sheet_1.pdf]

Supplementary Material

1.1 Supplementary Figures

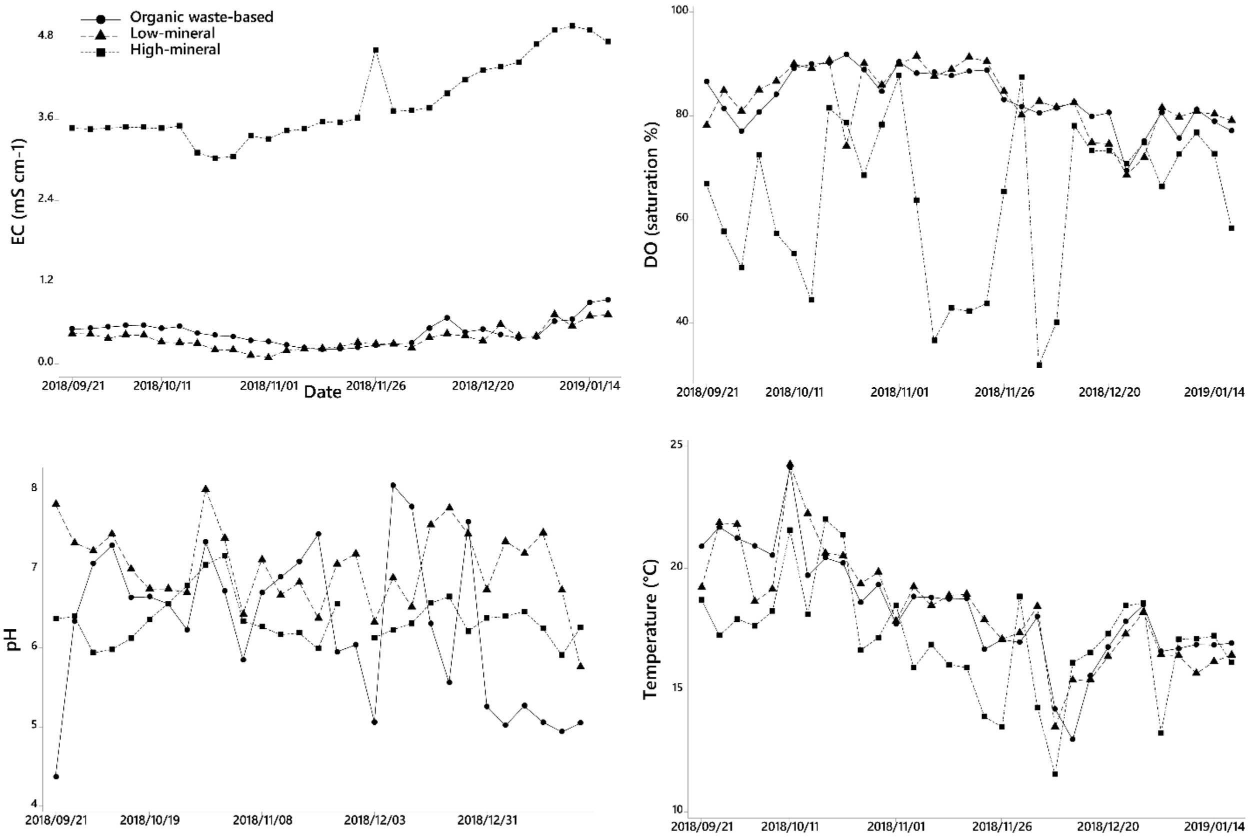

Suppl. Figure 1. Dynamic of physiochemical parameters of three treatments during whole cultivation period. ORP, oxidation reduction potential, DO, dissolved oxygen; EC, electrical conductivity. Individual measurements were performed twice a week, n = 28-31

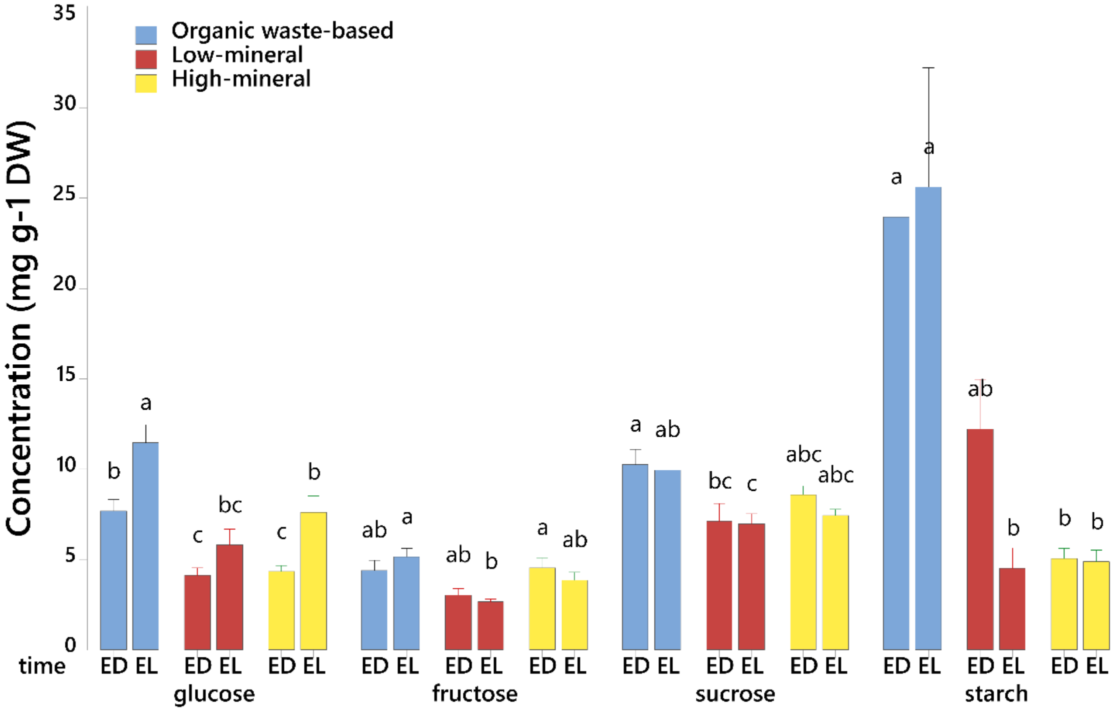

Suppl. Figure 2. Content of sugars in leaves of plants grown with different fertilizers. ED, end of dark; EL, end of light. Mean concentrations of individual anions are shown in mg·g^{-1} DW ± SD. Different letters indicate statistically significant differences at p = 0.05, n = 4-5.

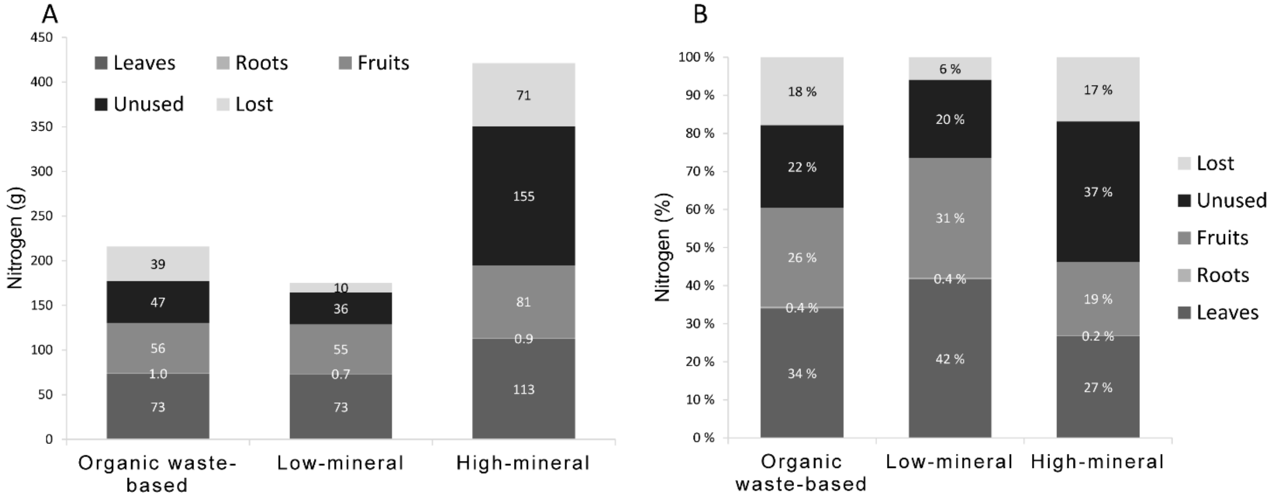

Suppl. Figure 3. Absolute and relative distribution of total nitrogen in different treatments. Distribution of N was estimated based on the total plant biomass, the amount of added fertilizer, the initial and final volume of nutrient solution, and the amount of nitric acid added for pH correction. It was assumed that N is 2% of fruits, roots and stems DW, content of N in leaves was 2.8, 2.2 and 2.7 % of DW for organic waste-based, low-, and high-mineral treatments, correspondingly. The estimation does not include amount of organic N in the digestate. Lost N corresponds to volatilization of N, spills and other undefined losses.

1.2 Supplementary Tables

Suppl. Table 1. Initial physiochemical parameters and ionic composition of the tested treatments. All measurements were done for samples obtained on 25.09.2018. ORP, oxidation reduction potential, DO, dissolved oxygen; EC, electrical conductivity.

Fertilizer	Organic	Low-Mineral	High-Mineral
	Physiochemical parameters		
Temperature (°C)	21.7	21.9	17.2
ORP (mV)	274	240	272
DO (% saturation)	81.4	84.9	57.7
pH	6.3	7.3	6.4
EC ($mS \cdot cm^{-1}$)	0.52	0.44	3.45
	Cations ($mmol \cdot L^{-1}$)		

Na^+	0.76	0.22	0.27
NH_4^+	0.02	0.00	1.25
K^+	1.45	1.23	9.81
Ca^{2+}	0.45	0.52	4.36
Mg^{2+}	0.25	0.22	2.63
Anions ($mmol \cdot L^{-1}$)			
Cl^-	0.93	0.57	0.15
NO_2^-	0.02	0.03	0.02
NO_3^-	2.05	1.49	21.76
PO_4^{3-}	0.50	0.44	1.26
SO_4^{2-}	0.27	0.18	2.08

Suppl. Table 2. Ionic composition of tomato fruits from plans cultivated on different fertilizers. Mean concentrations of individual ions are shown in $mmol \cdot L^{-1} \pm SD$. Different letters indicate statistically significant differences at $p = 0.05$, $n = 9$.

Component	Organic	Low-Mineral	High-Mineral
Inorganic ions ($mmol \cdot L^{-1}$) n=9			
Na^+	2.3 ± 0.4 a	0.7 ± 0.1 b	0.3 ± 0.1 c
NH_4^+	0.5 ± 0.1 a	0.3 ± 0.2 b	0.4 ± 0.2 a
K^+	52.6 ± 2.5 a	52.1 ± 5.1 a	72.1 ± 2.5 b
Ca^{2+}	3.2 ± 0.1 a	3.2 ± 0.1 a	3.2 ± 0.2 a
Mg^{2+}	2.7 ± 0.2 a	2.7 ± 0.3 a	3.0 ± 0.2 b

Cl^-	13.7 ± 2.4 a	3.8 ± 0.6 b	3.0 ± 0.4 b
NO_3^-	0.0 ± 0.1 a	0.8 ± 0.3 b	0.4 ± 0.3 a
PO_4^{3-}	4.4 ± 0.3 a	6.7 ± 0.6 b	7.8 ± 0.5 c
SO_4^{2-}	0.2 ± 0.1 a	1.5 ± 0.2 b	1.7 ± 0.2 b

Suppl. Table 3. Ionic composition of xylem sap from plants cultivated on different fertilizers. Mean concentrations of individual ions are shown in mmol·L^{-1} ± SD. Different letters indicate statistically significant differences at p = 0.05, n = 4.

Component	Organic	Low-Mineral	High-Mineral
Inorganic ions (mmol·L^{-1}) n=4			
Na^+	2.04 ± 0.37 a	0.25 ± 0.07 b	0.11 ± 0.01 b
NH_4^+	5.3 ± 0.6 a	0.6 ± 0.1 b	0.8 ± 0.1 b
K^+	16.6 ± 1.2 a	21.6 ± 1.9 b	17.5 ± 1.8 a
Ca^{2+}	4.7 ± 0.9 a	7.2 ± 0.3 b	13.1 ± 1.7 c
Mg^{2+}	1.9 ± 0.6 a	1.8 ± 0.1 a	4.1 ± 0.7 b
Cl^-	2.3 ± 0.2 a	1.2 ± 0.1 b	0.5 ± 0.1 c
NO_3^-	26.1 ± 2.4 a	26.8 ± 4.4 a	31.9 ± 4.4 a
PO_4^{3-}	1.9 ± 0.4 a	3.4 ± 0.6 a	5.9 ± 1.4 b
SO_4^{2-}	1.1 ± 0.3 a	3.3 ± 0.6 a	5.7 ± 1.2 a

Suppl. Table 4. Metabolites identified in xylem sap of plants grown on different fertilizers. Identification is performed in MSDIAL, statistics performed with MetaboAnalyst 5.0 (https://www.metaboanalyst.ca/). Concentrations are shown as IQF filtered mean peak height values,

and sorted by FDR-value. OWB, Organic waste-based; LM, Low-mineral; HM, High-mineral treatments.

Compound name	Organic	Low-Mineral	High-Mineral	f.value	p.value	-log10(p)	FDR	Fisher's LSD
Xylonic acid	2356.235	1226.938	3971.813	84.949	0.000	5.843	0.000	OWB - LM; LM - OWB; LM - LM
Ribose	4666.825	2272.060	9663.965	42.259	0.000	4.575	0.002	OWB - LM; LM - OWB; LM - LM
Putrescine	12084.481	1089.497	3570.560	20.672	0.000	3.365	0.017	OWB - LM; OWB - LM; LM - LM
Quinic acid	1873.247	957.591	5460.950	16.409	0.001	3.002	0.024	OWB - LM; LM - OWB; LM - LM
Malic acid	1371316.663	819863.188	572208.438	16.150	0.001	2.978	0.024	OWB - LM; OWB - LM; LM - LM
Valine	56363.668	35131.980	69641.915	13.510	0.002	2.710	0.037	OWB - LM; OWB - LM; LM - LM
Arginine	8404.470	7146.266	22139.410	11.881	0.003	2.525	0.049	OWB - LM; LM - OWB; LM - LM
glycero3-galactoside	249.828	19.931	554.906	11.238	0.004	2.447	0.051	OWB - LM; LM - OWB; LM - LM
Fucose	7562.060	3180.028	20963.575	9.328	0.006	2.194	0.071	OWB - LM; LM - OWB; LM - LM
citraconic acid	3443.225	3449.835	818.907	9.306	0.006	2.191	0.071	OWB - LM; OWB - LM; LM - LM
Glucuronate	3094.466	1284.607	9701.450	9.113	0.007	2.163	0.071	OWB - LM; LM - OWB; LM - LM
Lyxose	3016.944	1276.413	6332.897	8.882	0.007	2.130	0.071	OWB - LM; LM - OWB; LM - LM
asparagine	625794.548	335492.250	285900.026	8.387	0.009	2.056	0.072	OWB - LM; OWB - LM; LM - LM
Iditol	95607.268	49154.002	28278.545	8.082	0.010	2.009	0.072	OWB - LM; OWB - LM; LM - LM
3-Aminopropionitrile	10097.571	8444.838	2954.116	8.022	0.010	2.000	0.072	OWB - LM; OWB - LM; LM - LM
Fumaric acid	10101.036	8449.083	2942.878	8.013	0.010	1.999	0.072	OWB - LM; OWB - LM; LM - LM
Pyrophosphoric acid	6055162.750	7094799.750	8282567.500	7.731	0.011	1.954	0.074	LM - OWB; LM - OWB; LM - LM
Threonic acid	7278.777	3363.119	2374.619	7.605	0.012	1.934	0.074	OWB - LM; OWB - LM; LM - LM
Isoleucine	59290.120	42812.048	82739.035	7.306	0.013	1.885	0.078	OWB - LM; LM - OWB; LM - LM
Alanine	13113.566	40464.675	17002.954	7.068	0.014	1.845	0.078	LM - OWB; LM - OWB; LM - LM
phosphate	6101859.938	7141526.438	8329324.688	7.041	0.014	1.841	0.078	LM - OWB; LM - OWB; LM - LM
galactinol	3.261	3.691	1343.575	6.895	0.015	1.816	0.078	LM - OWB; LM - OWB; LM - LM
Galacturonic acid	5583.973	1233.769	4438.863	6.830	0.016	1.805	0.078	OWB - LM; OWB - LM; LM - LM
Hydroxyproline	826.432	101.483	771.210	6.688	0.017	1.780	0.078	OWB - LM; LM - OWB; LM - LM
propane-1,3-diol	8798.750	504.620	58100.550	6.641	0.017	1.772	0.078	OWB - LM; LM - OWB; LM - LM
glucose-1-phosphate	4867.329	5539.080	14629.516	6.407	0.019	1.730	0.082	LM - OWB; LM - OWB; LM - LM
a-Ketoglutaric acid	14942.050	8393.191	6988.910	6.278	0.020	1.707	0.083	OWB - LM; OWB - LM; LM - LM
sucrose	66416.728	13422.943	75286.748	6.220	0.020	1.696	0.083	OWB - LM; LM - OWB; LM - LM
Serine	20745.058	47289.870	20641.245	5.843	0.024	1.626	0.094	LM - OWB; OWB - LM; LM - LM
Lysine	179272.163	133889.038	351247.663	5.518	0.027	1.564	0.105	OWB - LM; LM - OWB; LM - LM
Allantoin	36712.410	13609.584	58558.285	5.322	0.030	1.525	0.111	OWB - LM; LM - OWB; LM - LM

Fructose	28132.562	5364.657	31417.158	5.244	0.031	1.510	0.111	OWB - LM; LM - OWB; LM - LM
isothreonic acid	1287.166	827.822	1832.588	4.739	0.039	1.406	0.137	OWB - LM; LM - OWB; LM - LM
allantoic acid dehydrated	1747.381	498.913	2306.429	4.575	0.043	1.371	0.144	OWB - LM; LM - OWB; LM - LM
itaconic acid	2229.797	2342.454	830.122	4.302	0.049	1.311	0.161	LM - OWB; OWB - LM; LM - LM
Myo-Inositol	107084.323	146567.450	235795.750	4.011	0.057	1.246	0.181	LM - OWB; LM - OWB; LM - LM
1-Amino-1-cyclopentanecarboxylic acid	17260.614	48825.158	10821.086	3.939	0.059	1.229	0.181	LM - OWB; OWB - LM; LM - LM
Mannose	5119.424	752.581	5580.769	3.830	0.063	1.203	0.181	OWB - LM; LM - OWB; LM - LM
Succinic acid	173526.410	150667.313	101125.713	3.780	0.064	1.192	0.181	OWB - LM; OWB - LM; LM - LM
5-Oxoproline	210413.700	175417.375	106085.973	3.772	0.065	1.190	0.181	OWB - LM; OWB - LM; LM - LM
Methionine	210382.088	175398.288	106066.135	3.771	0.065	1.190	0.181	OWB - LM; OWB - LM; LM - LM
Tyramine	2842.078	1004.594	4169.782	3.706	0.067	1.174	0.183	OWB - LM; LM - OWB; LM - LM
UDP-glucuronic acid	224.144	276.675	1099.160	3.597	0.071	1.148	0.188	LM - OWB; LM - OWB; LM - LM
gamma-aminobutyric acid	277359.772	47773.863	668501.190	3.530	0.074	1.132	0.188	OWB - LM; LM - OWB; LM - LM
N-Acetylputrescine	20262.790	16526.618	9265.276	3.513	0.075	1.128	0.188	OWB - LM; OWB - LM; LM - LM
Lactic acid	15894.502	529.263	89428.216	3.493	0.075	1.123	0.188	OWB - LM; LM - OWB; LM - LM
glucose	30365.582	3958.113	29483.425	3.403	0.079	1.101	0.194	OWB - LM; LM - OWB; LM - LM
butyrolactam	1092.265	707.128	4608.847	3.283	0.085	1.071	0.204	LM - OWB; LM - OWB; LM - LM
aconitic acid	1071.963	1061.525	3534.041	3.045	0.098	1.010	0.229	OWB - LM; LM - OWB; LM - LM
Tryptophan	698.194	274.006	644.600	2.660	0.124	0.908	0.285	OWB - LM; OWB - LM; LM - LM
Tyrosine	111644.575	69827.825	97688.575	2.603	0.128	0.892	0.289	OWB - LM; OWB - LM; LM - LM
Ornithine	129487.608	56554.455	88932.903	2.520	0.135	0.869	0.298	OWB - LM; OWB - LM; LM - LM
Glutamate	37316.445	17414.023	45020.430	2.496	0.137	0.862	0.298	OWB - LM; OWB - LM; LM - LM
O-Acetyserine	4919.628	3544.722	2985.565	2.416	0.145	0.840	0.308	OWB - LM; OWB - LM; LM - LM
citric acid	85560.195	36484.620	63910.570	2.378	0.148	0.829	0.310	OWB - LM; OWB - LM; LM - LM
pyruvic acid	1759.410	488.285	153.720	1.951	0.198	0.704	0.394	OWB - LM; OWB - LM; LM - LM
Benzoic acid nRT	338.447	101.962	388.923	1.939	0.199	0.700	0.394	OWB - LM; OWB - LM; LM - LM
dehydroascorbic acid	427.253	174.706	700.300	1.935	0.200	0.699	0.394	OWB - LM; LM - OWB; LM - LM
proline	24073.835	27300.538	57161.913	1.920	0.202	0.694	0.394	LM - OWB; LM - OWB; LM - LM
Glycerol	767570.290	1262417.563	1788963.813	1.868	0.210	0.678	0.396	LM - OWB; LM - OWB; LM - LM
Cadaverine	1778.307	1576.916	2711.447	1.864	0.210	0.677	0.396	OWB - LM; LM - OWB; LM - LM
capric acid	224.890	412.265	73.380	1.795	0.221	0.656	0.410	LM - OWB; OWB - LM; LM - LM
DIBUTYL PHTHALATE	1823.528	926.154	1767.216	1.748	0.228	0.641	0.414	OWB - LM; OWB - LM; LM - LM
N-acetylornithineb	562.831	280.675	526.113	1.736	0.230	0.638	0.414	OWB - LM; OWB - LM; LM - LM
levoglucosan	126.452	137.679	655.225	1.705	0.236	0.628	0.417	OWB - LM; LM - OWB; LM - LM

urea	1184.828	1408.938	4095.766	1.640	0.247	0.607	0.428	LM - OWB; LM - OWB; LM - LM
hydroxylamine	490.538	614.243	93.695	1.611	0.252	0.598	0.428	OWB - LM; OWB - LM; LM - LM
tranexamic acid	613.935	320.313	184.098	1.602	0.254	0.595	0.428	OWB - LM; OWB - LM; LM - LM
Stearic acid	67296.750	59658.750	40135.750	1.587	0.257	0.590	0.428	OWB - LM; OWB - LM; LM - LM
1,2-BENZENEDICARBOXYLIC ACID ISOPROPYETHYLHEXYL ESTER	1743.528	1255.372	1666.950	1.543	0.265	0.576	0.436	OWB - LM; OWB - LM; LM - LM
Xylitol	190272.488	158055.738	268145.738	1.480	0.278	0.556	0.450	OWB - LM; LM - OWB; LM - LM
Mesaconic acid	117.683	172.647	431.131	1.441	0.287	0.543	0.458	LM - OWB; LM - OWB; LM - LM
Glyceric acid	3356.085	316.475	5260.521	1.380	0.300	0.523	0.468	OWB - LM; OWB - LM; LM - LM
beta-Cyano-alanine	74913.225	48088.725	51619.225	1.375	0.301	0.521	0.468	OWB - LM; OWB - LM; LM - LM
O-Phosphoethanolamine	3678.503	4628.129	6282.863	1.303	0.318	0.497	0.488	LM - OWB; LM - OWB; LM - LM
Picolinic acid	1384.997	1055.357	556.326	1.248	0.332	0.478	0.500	OWB - LM; OWB - LM; LM - LM
Myristic acid	2880.553	3389.913	2776.085	1.239	0.335	0.475	0.500	LM - OWB; OWB - LM; LM - LM
Threonine	131000.438	126695.523	194760.363	1.213	0.342	0.466	0.504	LM - OWB; LM - OWB; LM - LM
N-OCTYLBISTRIMETHYLSILYLAMINE	192.775	427.100	52.700	1.173	0.353	0.453	0.513	LM - OWB; OWB - LM; LM - LM
diethyl phthalate	2235.724	2204.703	5672.263	1.098	0.374	0.427	0.533	LM - OWB; LM - OWB; LM - LM
Phenylalanine	47172.425	54886.800	59624.485	1.095	0.375	0.426	0.533	LM - OWB; LM - OWB; LM - LM
Glycolic acid	2708.883	2923.605	709.035	1.044	0.391	0.408	0.543	LM - OWB; OWB - LM; LM - LM
Maleic acid	42497.933	52431.698	28991.295	1.042	0.392	0.407	0.543	OWB - LM; OWB - LM; LM - LM
Ethanolamine	17009.525	14269.300	74796.000	0.959	0.419	0.378	0.574	OWB - LM; LM - OWB; LM - LM
Lauric acid	678.135	645.025	603.228	0.910	0.437	0.360	0.581	LM - OWB; OWB - LM; LM - LM
leucine	225667.163	173725.163	227517.163	0.908	0.437	0.359	0.581	OWB - LM; OWB - LM; LM - LM
1-TRIMETHYLSILOXYCYCLOPENTENE	6760.045	4849.780	4230.045	0.901	0.440	0.357	0.581	OWB - LM; OWB - LM; LM - LM
Shikimic acid	5707.938	5796.047	15390.081	0.882	0.447	0.350	0.584	OWB - LM; LM - OWB; LM - LM
ISOBUTYRIC ACID	5985.033	7690.933	10499.360	0.789	0.483	0.316	0.620	OWB - LM; LM - OWB; LM - LM
Acetylsalicylic acid	170.431	85.594	258.548	0.785	0.485	0.314	0.620	OWB - LM; LM - OWB; LM - LM
Gallic acid	337.631	340.538	609.928	0.757	0.497	0.304	0.628	LM - OWB; LM - OWB; LM - LM
Palmitic acid	51219.735	36035.200	19090.075	0.570	0.585	0.233	0.731	LM - OWB; OWB - LM; LM - LM
Diethanolamine	199.435	66.431	173.775	0.533	0.604	0.219	0.731	OWB - LM; OWB - LM; LM - LM
DIOCTYL PHTHALATE	909.997	965.841	1012.466	0.531	0.605	0.218	0.731	LM - OWB; OWB - LM; LM - LM
Caffeic acid	411.756	517.297	1256.422	0.525	0.608	0.216	0.731	LM - OWB; LM - OWB; LM - LM

Ribitol	97302.348	68665.348	215110.130	0.523	0.610	0.215	0.731	OWB - LM; LM - OWB; LM - LM
Nonanoric acid	2157.989	3247.410	2191.784	0.475	0.637	0.196	0.755	LM - OWB; OWB - LM; LM - LM
Phthalic acid	3956.550	2053.800	3858.113	0.387	0.690	0.161	0.809	OWB - LM; OWB - LM; LM - LM
glutamine	602797.241	552831.276	618546.491	0.373	0.699	0.156	0.812	OWB - LM; OWB - LM; LM - LM
Glycine	3800.297	4115.219	4538.750	0.357	0.709	0.149	0.816	LM - OWB; LM - OWB; LM - LM
Pentadecanoic acid	806.181	893.400	876.666	0.343	0.718	0.144	0.818	LM - OWB; OWB - LM; LM - LM
2-methynonadecane	161.070	114.505	510.820	0.325	0.730	0.136	0.823	OWB - LM; LM - OWB; LM - LM
1-Aminocyclopropane-1-carboxylic acid	5839.752	2450.053	5115.101	0.304	0.745	0.128	0.832	OWB - LM; OWB - LM; LM - LM
D2,3-BISTRIMETHYLSILYLOXY-PROPIONIC ACID	2324.972	976.004	3313.847	0.293	0.753	0.123	0.832	OWB - LM; OWB - LM; LM - LM
uracil	452.060	466.532	526.313	0.182	0.837	0.077	0.916	LM - OWB; LM - OWB; LM - LM
methylamine	75754.605	102060.838	124385.388	0.139	0.872	0.060	0.946	LM - OWB; LM - OWB; LM - LM
Gluconic acid	15788.159	8193.412	15169.972	0.092	0.913	0.040	0.981	OWB - LM; LM - OWB; LM - LM
1,2-DIACETOXYETHANE	617496.010	625115.010	889155.430	0.076	0.927	0.033	0.983	OWB - LM; LM - OWB; LM - LM
DIMETHYLHYDRAZONE BUTYRALDEHYDE	56105.423	60453.748	82714.805	0.070	0.933	0.030	0.983	OWB - LM; LM - OWB; LM - LM
ETHYL ETHOXYACETATE	523004.963	481185.963	664229.950	0.062	0.940	0.027	0.983	OWB - LM; LM - OWB; LM - LM
3-PENTANOL	521768.663	529920.163	688053.050	0.041	0.960	0.018	0.993	OWB - LM; LM - OWB; LM - LM
Methylmalonic acid	11615.653	9344.617	4798.165	0.028	0.972	0.012	0.993	LM - OWB; OWB - LM; LM - LM
Aspartic acid	144026.270	130815.729	223180.979	0.024	0.976	0.011	0.993	OWB - LM; LM - OWB; LM - LM
2-METHOXYPYRIDINE	243250.300	269891.250	310604.400	0.015	0.985	0.006	0.993	OWB - LM; LM - OWB; LM - LM
3-ISOPROPY6,10-DIMETHYLUNDECANE-2-OL	152.309	134.184	153.832	0.007	0.993	0.003	0.993	OWB - LM; LM - OWB; LM - LM
